# Accurate positioning of functional residues with robotics-inspired computational protein design

**DOI:** 10.1073/pnas.2115480119

**Published:** 2022-03-07

**Authors:** Cody Krivacic, Kale Kundert, Xingjie Pan, Roland A. Pache, Lin Liu, Shane O Conchúir, Jeliazko R. Jeliazkov, Jeffrey J. Gray, Michael C. Thompson, James S. Fraser, Tanja Kortemme

**Affiliations:** ^a^UC Berkeley–UCSF Graduate Program in Bioengineering, University of California, San Francisco, CA 94158;; ^b^Department of Bioengineering and Therapeutic Sciences, University of California, San Francisco, CA 94158;; ^c^Biophysics Graduate Program, University of California, San Francisco, CA 94158;; ^d^Program in Molecular Biophysics, Johns Hopkins University, Baltimore, MD 21218;; ^e^Department of Chemical and Biomolecular Engineering, Johns Hopkins University, Baltimore, MD 21218;; ^f^Quantitative Biosciences Institute, University of California, San Francisco, CA 94158

**Keywords:** computational protein design, structure prediction, design of function, Rosetta

## Abstract

Proteins achieve their complex functions, such as molecular recognition with high affinity and specificity, through intricate three-dimensional geometries in functional sites. To engineer new protein functions, accurate positioning of amino acid functional groups is therefore critical but has remained difficult to achieve by computational methods because of current limitations in the design of new conformations with arbitrary user-defined geometries. Here, we introduce two computational methods capable of generating and predicting new local protein geometries: fragment kinematic closure (FKIC) and loophash kinematic closure (LHKIC). FKIC and LHKIC integrate two approaches: robotics-inspired kinematics of protein conformations and insertion of peptide fragments. We show that FKIC and LHKIC predict native-like conformations at atomic accuracy and with up to 140-fold improvements in sampling efficiency over previous approaches. We then use these methods to create a design protocol, pull into place (PIP), to position functionally important side chains via design of backbone conformations. We validate PIP by remodeling a sizeable active site region in an enzyme and confirming the engineered new conformations of two designs with crystal structures. The described methods can be applied broadly to the design of user-defined geometries for new protein functions.

## Significance

Computational protein design promises to advance applications in medicine and biotechnology by creating proteins with many new and useful functions. However, new functions require the design of specific and often irregular atom-level geometries, which remains a major challenge. Here, we develop computational methods that design and predict local protein geometries with greater accuracy than existing methods. Then, as a proof of concept, we leverage these methods to design new protein conformations in the enzyme ketosteroid isomerase that change the protein’s preference for a key functional residue. Our computational methods are openly accessible and can be applied to the design of other intricate geometries customized for new user-defined protein functions.

Advances in computational protein design (1, 2) promise to create new proteins to impact current and future challenges in biotechnology and medicine. Computationally designed proteins already enable important applications as modular sense/response systems to control precise biological responses (3), as nanoparticles for potent protein vaccines (4), and as protein therapeutics with minimal side effects (5). However, while new “idealized” protein structures consistent primarily of regular secondary structure elements connected by short loops can now often be designed rather robustly (6), the design of new functions remains more difficult (2, 7).

A key challenge lies in the difficulty of designing the fine-tuned protein geometries necessary for function with atomic accuracy. Many functions involve considerable deviations from the idealized highly stable de novo designed structures that are much easier to design (8, 9). Further difficulties arise both from the small energy gaps between functional and nonfunctional conformations (10) and the formidable problem of sampling the enormous space of possible sequence/structure combinations (11). Taken together, these issues complicate the accurate positioning of amino acid functional groups for many applications involving specific molecular recognition.

Accurate positioning of key amino acid side-chain functional groups by computational design is particularly challenging in cases where the desired geometry cannot be achieved by simply placing new side chains on an existing or slightly modified backbone, but instead requires generation and design of substantially altered backbone conformations. Despite the importance of this capability for designing proteins with new user-defined functions, as well as prior work on local alterations of active sites (12, 13), this problem has remained generally unsolved.

Here, we describe and experimentally validate an approach for designing substantially altered protein conformations that accurately position user-defined functional groups in proteins, called pull into place (PIP). The PIP protocol has three steps: 1) generation of new backbone conformations, in which functional groups of interest are gently pulled toward their desired positions using harmonic restraints; 2) sequence design using fixed-backbone side-chain optimizations with the same restraints; and 3) structure prediction using unrestrained flexible-backbone simulations to identify designs predicted to adopt the desired new backbone conformation. We demonstrate that PIP is capable of accurately placing side chains and designing the required considerable alterations of the protein backbone by solving crystal structures of two designs. Detailed characterization of one successful design reveals a robustness to mutation, suggesting that multiple interactions contribute to the conformation of the remodeled region. The design methods described here advance the engineering of new proteins by allowing the accurate positioning of functional groups critical for many aspects of protein function, such as specific recognition of binding partners.

## Results

**Fig. 1.**
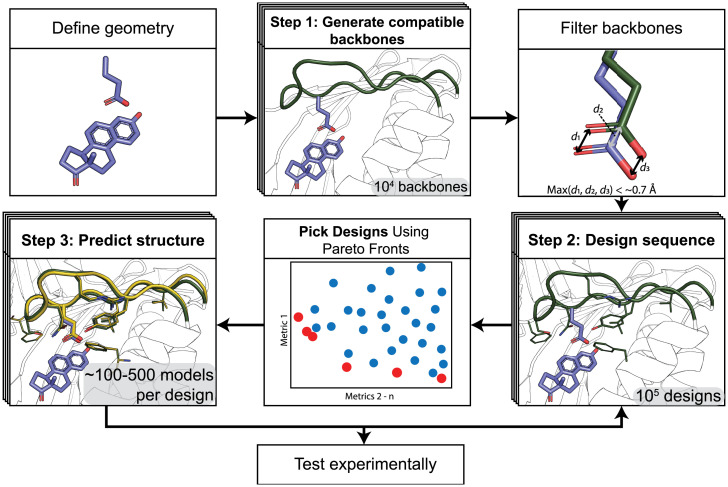
Steps of the PIP protocol. *Top Left*: functional geometry is defined. *Top Middle*: New backbone conformations (green) are generated to satisfy the geometric restraints. *Top Right*: Backbones are filtered based on their ability to satisfy the geometric restraints. d1, d2, and d3 refer to the distances of the atoms in a positioned carboxyl group to their defined ideal positions. *Bottom Right*: Sequences are designed to stabilize the de novo backbone. *Bottom Middle*: Designs are selected based on multiple computational quality metrics using Pareto fronts (*SI Appendix*, *SI Methods*). Red: Pareto-efficient designs; blue: other designs. *Bottom Left*: For selected sequences, Rosetta structure prediction method are applied to predict the lowest-energy structure (yellow). Illustrations use the KSI model system detailed in Fig. 3.

We set out to develop a method (PIP) to accurately position amino acid functional groups in proteins by designing new local backbone geometries. The PIP algorithm required three components (Fig. 1): 1) a method to generate designable backbone conformations that could precisely position defined functional groups, 2) a way to stabilize these new backbone and side-chain conformations by finding sequences optimal for the desired structure, and 3) a method to predict the new conformation given a sequence to assess whether the desired structure is also optimal for the designed sequence.

We first describe two improved computational methods, fragment kinematic closure (FKIC) and loophash kinematic closure (LHKIC), to generate new backbone conformations (step 1) and to predict their structures accurately given designed sequences (step 3). These methods are particularly suited to problems for which 1) target structures do not exclusively adopt regular secondary structure geometries, 2) there are no protein homologs that can be used as templates for modeling, and 3) there are no multiple sequence alignments to guide current deep learning structure prediction methods (14, 15), since we aim to design new structures and sequences. We then describe the application of the entire PIP protocol in the program Rosetta to a design problem in which we reshape the backbone geometry of a model protein, ketosteroid isomerase (KSI), to replace a functional aspartate with a glutamate residue (not found in any KSI homologs) such that the carboxyl groups align. We chose this design problem as a proof of concept because it presents a particularly challenging positioning problem that cannot be solved with (near–) fixed-backbone design, for which no solution was known in a homologous protein and that requires accuracy on the length scale of a carbon–carbon bond.

### FKIC and LHKIC Algorithms.

**Fig. 2.**
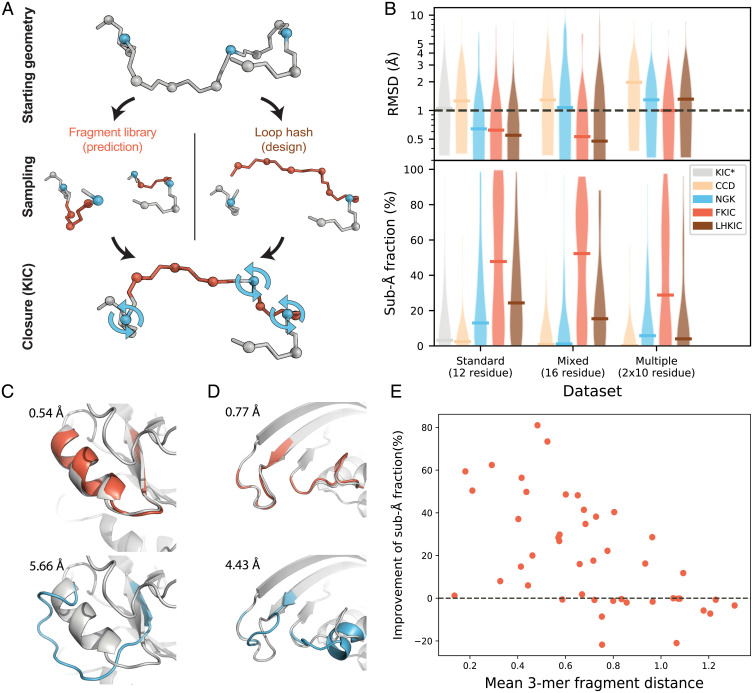
FKIC improves prediction of conformations of local backbone segments. (*A*) Individual FKIC/LHKIC move. Three Cα atoms (blue) on the target segment to be modeled (gray) are picked randomly as pivots. Fragment insertion (FKIC) or loop hash (LHKIC) is applied to sample torsion degrees of freedom at nonpivot atoms (red), which breaks the chain. The KIC algorithm is then used to close the chain by determining appropriate values for the pivot torsions. (*B*) Comparison of performance of different methods for three datasets: 1) the Standard dataset described in ref. 17 and two new sets, 2) a “Mixed Segment” dataset with 30 16-residue regions that contain both loops and segments of regular secondary structure and 3) a “Multiple Segments'' dataset of 30 cases with two separate 10-residue regions that are interacting. KIC (17): gray; CCD (24): orange; NGK (26): blue; FKIC: red; LHKIC: brown. *Upper*: violin plot of RMSD of lowest energy (best) model across each dataset. Horizontal bars indicate the median lowest-energy RMSD. FKIC is the only method that provides predictions with atomic accuracy (≤1 Å median RMSD) for all datasets. *Lower*: violin plot of fraction of predicted models in each dataset that have subångstrom accuracy. FKIC leads to considerable improvements over previous methods. Asterisk indicates data from ref. 42; all other simulations were run with the ref2015 Rosetta energy function (21); methods using fragments (CCD and FKIC) used identical fragment libraries that excluded fragments from structural homologs to the target proteins. (*C* and *D*) FKIC accurately predicts geometries from sequence in which the previous state-of-the-art method, NGK, fails. Shown are examples from the Mixed Segment (*C*) and Multiple Segments dataset (*D*). Experimentally determined structures: gray; predictions from FKIC: red, *Top*; predictions from NGK: blue, *Bottom*. RMSDs to the experimentally determined structures are given in each panel in Å. (*E*) The fraction of subångstrom predictions is negatively correlated with the mean 3-mer fragment distance (*Methods*). Each data point represents a protein from the Standard 12-residue dataset.

FKIC and LHKIC integrate two concepts that have separately led to considerable advances in protein modeling: sampling preferred combinations of backbone torsions from fragments of proteins in the Protein Data Bank (PDB) (16) and improved sampling of segments without regular secondary structure or template information with an inverse kinematic closure algorithm termed “KIC” (17) borrowed from the field of robotics (18). KIC determines “mechanically accessible” conformations for internal protein segments of given lengths by sampling the φ/ψ torsion degrees of freedom in the segment. In each KIC move, three Cα atoms of an N-residue segment are designated as pivots, leaving N − 3 nonpivot Cα atoms. In the standard implementation of KIC in Rosetta (17), nonpivot torsions are sampled from a residue type-specific Ramachandran map. In the FKIC method used here (see *Methods* for details), nonpivot degrees of freedom are taken from peptide fragments that are picked from the PDB using the sequence of the target segment (Fig. 2 *A*, *Left*) (19); KIC is then used to determine the values of the pivot torsions that close the resulting chain break. We reasoned that FKIC would combine the improved prediction accuracy of KIC demonstrated previously (17) with improved sampling efficiency because of the reduction of degrees of freedom by using coupled torsion angles from fragments (in contrast to sampling all nonpivot torsions independently from Ramachandran space as in KIC, which is unlikely, for example, to sample regular secondary structures).

To mimic a design case in which the sequence of the modeled segment is not known a priori, we also developed a variation on the method, LHKIC, which uses the loophash protocol (20) to pick fragments that simultaneously sample structures and sequences of the target segments. The loophash protocol uses the 6D transformation between the residue before the first pivot and the residue after the last pivot as a query key to find peptide fragments from the PDB that approximately close the gap between these two residues (Fig. 2 *A*, *Right*). After insertion of a fragment, KIC determines the pivot torsions that close the gap. For design cases, LHKIC can optionally mutate remodeled residues to the amino acids from the inserted fragment to improve local sequence-structure compatibility. Individual FKIC or LHKIC sampling moves (Fig. 2*A*) are then followed by optimization of side-chain conformations in and around the altered backbone region and integrated into a Monte Carlo minimization protocol (*SI Appendix*, Fig. 1); sampled conformations are evaluated with Rosetta’s all-atom energy function (21, 22).

### Local Structure Prediction Performance.

We tested the ability of FKIC to recapitulate the local conformations of protein segments, given their sequences, on three benchmark sets. The first is a benchmark of 45 12-residue loops (23) previously used to evaluate KIC (17) (“Standard” set), to enable comparisons with published work. We also used two new sets representing more challenging problems closer to design applications: a set of 30 16-residue segments in which each segment contains both regular secondary structure elements and loop regions (“Mixed Segment” set) and a set of 30 pairs of interacting 10-residue segments (“Multiple Segments” set). As controls, we applied methods that use KIC and fragment insertion (closed cyclic descent [CCD]) (24, 25) alone to the same datasets using an otherwise identical protocol in Rosetta. We used two performance metrics: The first quantifies prediction accuracy by determining the root mean-square deviation (RMSD) of the model with the lowest (best) predicted Rosetta energy to each native structure and then taking the median RMSD value across each dataset. The second metric quantifies sampling efficiency by measuring the fraction of native-like (correct) models generated for each protein case, for which native-like is defined as <1 Å (“subångstrom”) RMSD to the native structure, and again taking the median for each dataset (*Methods*; *SI Appendix*, Table 1A).

The Rosetta KIC method had previously been shown (17) to be comparable to a state-of-the-art molecular mechanics method (23). The next-generation KIC (NGK) update (26) led to improved performance over KIC and had comparable performance to GalaxyLoop-PS2 (27), RCD+ (28), Sphinx (29), LEAP (30), and FREAD (31, 32) when tested on identical datasets. Here, we show that FKIC improves structure prediction accuracy over CCD, KIC, and NGK, with the largest changes for the two new datasets (Fig. 2 *B*, *Top*; *SI Appendix*, Table 1A). On the 16-residue Mixed Segment dataset, which tests the ability of FKIC to predict conformations of protein segments with arbitrary secondary structure composition, the median accuracy improved to 0.53 Å RMSD with FKIC compared to 1.29 Å and 1.07 Å with CCD and NGK alone, respectively. For the Multiple Segments dataset, which tests the ability of FKIC to predict conformations of discontinuous interacting segments, FKIC was the only method that yielded atomic (1-Å) median accuracy, compared to 1.97 Å and 1.29 Å with CCD and NGK alone, respectively (Fig. 2 *B*, *Top*; *SI Appendix*, Table 1A). Representative examples for which FKIC correctly predicted protein conformations while NGK failed are shown for the Mixed Segment and Multiple Segments datasets in Fig. 2 *C* and *D*, and details are given in *SI Appendix*, Tables 2 and 3. The improvements on the Standard dataset were smaller (median RMSD was 0.62 Å with FKIC compared to 0.64Å for NGK; *SI Appendix*, Table 1A), but for 35/45 proteins, FKIC finds lower energy structures than NGK (*SI Appendix*, Table 4). Cases in which FKIC predictions did not lead to the identification of subångstrom-accuracy lowest-scoring models can be attributed to both sampling and energy function limitations (*SI Appendix*, Table 5, Fig. 2 and Note 1).

FKIC also considerably improved sampling efficiency, which we quantified by how frequently FKIC generated conformations that are <1 Å RMSD from the crystallographic conformation (Fig. 2 *B*, *Bottom*). For the Mixed Segment set, the median fraction of subångstrom predictions for FKIC was 52.3%, which was 45- and 105-fold higher than for NGK and CCD, respectively. For the Multiple Segments dataset, the median fraction of subångstrom predictions was 28.5% with FKIC, which was fivefold higher than with NGK (5.5%) and 143-fold higher (0.2%) than with CCD (*SI Appendix*, Table 1A). In several cases, FKIC was able to find correct solutions for even larger conformational sampling problems such as a set with two interacting 12-residue segments (*SI Appendix*, Note 2 and Tables 6 and 7). These improvements in sampling efficiency are important in particular for design since they reduce the computational time needed to predict the conformation of a reshaped backbone segment, allowing for more designs to be evaluated.

We also tested the ability of LHKIC to predict local protein conformations on the three benchmark sets. LHKIC performed similarly to FKIC in terms of RMSD (Fig. 2 *B*, *Top*; *SI Appendix*, Table 1A and Note 3). However, in this structure prediction task, LHKIC sampling efficiency was lower than for FKIC (Fig. 2 *B*, *Bottom*) since LHKIC does not use information on the target sequence for picking fragments. LHKIC is therefore intended for design applications in which sequence and structure are sampled simultaneously, rather than for structure prediction tasks in which the sequence is known and fixed.

Overall, the improvement of the fraction of subångstrom predictions is negatively correlated with the mean 3-mer fragment distance from the native structure (Fig. 2*E* and *Methods*). This observation shows that high quality fragments focus the sampling on native-like conformations. While both CCD and FKIC sample from the same fragment set, FKIC performs considerably better (Fig. 2*B*). This difference between the two fragment-based structure prediction methods could at least partly be attributed to the fact that when CCD closes a chain break, it modifies all torsions along the inserted fragment, while KIC maintains more conformational information from the inserted fragment by only modifying the three pivot residues. While high-quality fragments could be derived from homologous structures, for both CCD and FKIC benchmark simulations we excluded fragments from homologs to test the ability to predict structures of regions for which there are no templates. However, we also repeated our simulations with fragments from homologs present in the database. As expected, both prediction accuracy and the median fraction of subångstrom predictions improved further when homologous structures are included in FKIC simulations (*SI Appendix*, Table 1B).

### Application of the PIP Protocol.

**Fig. 3.**
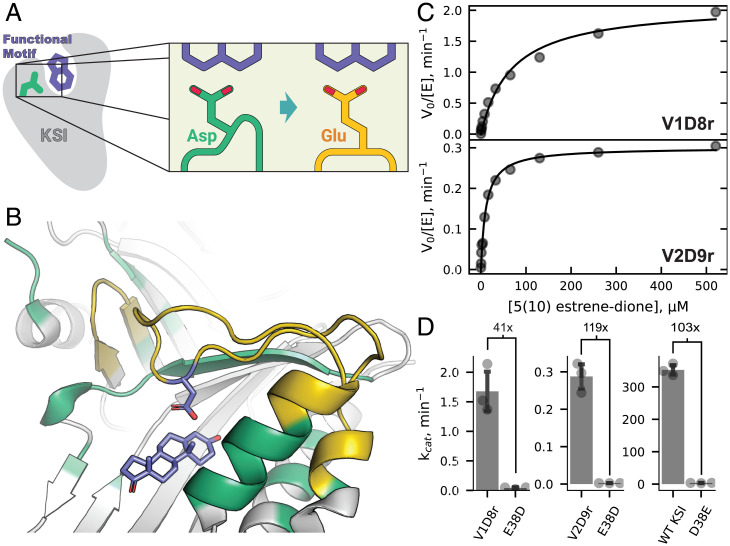
Functional characterization of designs V1D8r and V2D9r. (*A*) Schematic of design goal for KSI. Green: wild-type KSI with catalytic aspartate. Yellow: Designed KSI variant with reshaped active site to position the glutamate carboxyl group in place of the wild-type aspartate carboxyl group. (*B*) KSI wild-type structure (PDB 1QJG), showing the active site regions to be remodeled. Residues allowed to change identity (design) or conformation (repack) during the design process (PIP version 2) are shown in yellow or green, respectively, and static positions are shown in gray. (*C*) Representative Michaelis-Menten curves for design V1D8r (*Top*) or V2D9r (*Bottom*). (*D*) Bar plots showing the k_cat_ values of V1D8r (*Top*), V2D9r (*Middle*), or wild-type KSI (*Bottom*) and their E38D or D38E active site mutations. Values show the fold change in k_cat_ between the respective D/E active site residue pairs. SDs of independent triplicate experiments are shown as error bars, with individual measurements shown as points.

With improved methods for sampling and prediction of backbone conformations in hand, we set out to test the entire PIP protocol (Fig. 1) in a design application. We chose *Pseudomonas testosteroni* KSI as a model system (Fig. 3*A*). KSI uses a catalytic aspartate at position 38 to abstract a proton from a steroid substrate to catalyze an energetically favorable double-bond rearrangement. Here, we set out to replace aspartate 38 with glutamate while maintaining the precise placement of the side-chain carboxyl group (Fig. 3*A*) by reshaping a sizeable region of the protein backbone (11 to 12 residues; Fig. 3*B*). To test our designs before solving atomic-resolution structures, we reasoned that KSI activity provides a convenient way to estimate the accuracy of functional group positioning because KSI activity is sensitive to perturbations of the functional site geometry on the length scale of a carbon–carbon bond: With 5 (10)-estrene-3,17-dione as a substrate, mutating aspartate 38 in KSI to a glutamate reduces the protein’s k_cat_ by ∼103-fold (Table 1) (this value is similar to previous work that reported a reduction of 240-fold in the D38E mutant compared with wild-type; Ref. 33). This reduction in k_cat_ is attributed to the misplacement of the side-chain carboxyl group that is common to glutamate and aspartate because of the additional methylene group in the glutamate side chain. We note that the PIP design protocol is not geared toward optimizing catalytic activity, as the protocol does not specifically consider requirements of catalysis other than positioning of functional groups. However, enzyme activity is still a useful proxy to probe for accurate positioning when comparing aspartate to glutamate. Moreover, no known homologs of KSI contain a glutamate at the catalytic position (34). Thus, any designed solutions would be novel, and a fragment-based design protocol would not be able to rely on naturally occurring homologs that have already solved this particular problem.

**Table 1.** Kinetic parameters of WT KSI, WT D38E, and designs

Enzyme	k_cat_ (min^−1^)	K_M_ (μM)	k_cat_/K_M_ (μM^−1^ ⋅ min^−1^)
WT	350 ± 18	120 ± 32	2.9 ± 0.79
WT D38E	3.4 ± 0.50	37 ± 4.9	0.092 ± 0.018
V1D8r	1.7 ± 0.41	67 ± 15	0.025 ± 0.0084
V2D9r	0.29 ± 0.0040	9.0 ± 2.0	0.032 ± 0.0084

Ranges are based on the SD of three independent experiments. WT, wild type.

Our PIP design protocol for KSI (Fig. 1) proceeded in three steps: In step 1, we built 20,000 de novo backbone conformations that positioned the functional carboxyl group using harmonic coordinate restraints defined by the amide atoms of asparagine 38 (an inactivating mutation for the catalytic D38 that enables a transition state mimic to be crystallized) in PDB ID: 1QJG in place of the catalytic D38. We selected the 1,600 or 4,000 conformations (numbers are for two rounds of the protocol, see *Methods*) that best matched the desired geometry based on their restraint satisfaction, which we defined as the maximum distance of any restrained atom in the model to the atom’s ideal position (Fig. 1, *Top Right*).

In the second step, these reshaped backbone conformations were stabilized by redesigning the local environment, where all residues of the reshaped backbone segments as well as residues in the environment were redesigned using design methods in Rosetta (*SI Appendix*, *SI Methods*). This process resulted in 10 to 50 designs per input structure. We then selected 200 or 422 design models for structure prediction in step 3. These designs were selected based on how close the modeled catalytic residue carboxyl group atoms were to their desired positions and on several computational design quality metrics including Rosetta score terms, hydrogen bond satisfaction, and metrics for sequence-structure compatibility (see *Methods* for details).

While step 2 (design) aims to find sequences that are optimal for the targeted conformations, step 3 (structure prediction) aims to assess whether these sequences indeed fold into the targeted conformation (i.e., is the conformation also optimal given the sequence). Steps 2 and 3 were iterated to further optimize sequence-structure combinations. In particular, designed sequences that produced structure prediction models that correctly placed the functional carboxyl group but were not the lowest-scoring model generated by the structure prediction protocol were fed back to step 2 for further sequence optimization.

### Selection of Designed KSI Variants.

**Fig. 4.**
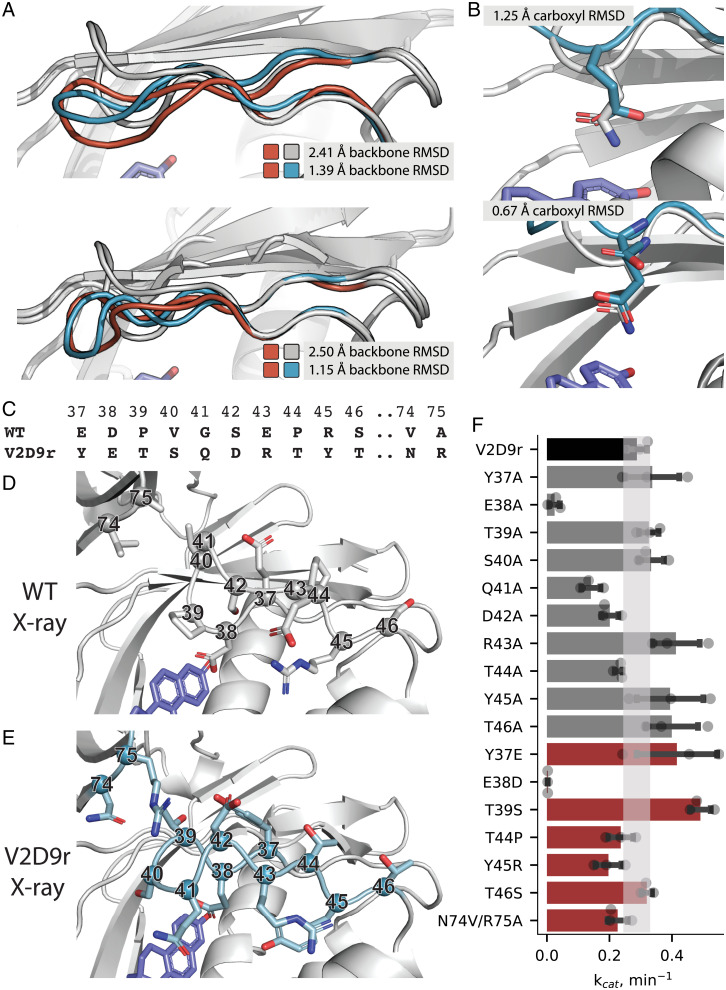
Structural characterization of designs V1D8r and V2D9r. (*A*) Overlay of wild-type KSI crystal structure (gray), lowest-energy predicted models for V1D8r (orange, *Top*) and V2D9r (orange, *Bottom*), and crystal structures for V1D8r (blue, *Top*) and V2D9r (blue, *Bottom*). (*B*) Crystal structure (blue) of V1D8r (*Top*) and V2D9r (*Bottom*) showing the catalytic glutamate’s placement relative to the amide in the KSI starting structure (PDB 1QJG) used to define the catalytic position (gray). RMSD values between compared structures are indicated in the different panels. (*C*–*F*) Mutational analysis of differences between wild-type KSI and design V2D9r: sequence alignment (*C*), comparison between the active site region in the crystal structures of wild-type KSI (*D*) and in design V2D9r (*E*), and (*F*) bar graph of k_cat_ values for design V2D9r (black), alanine scan mutants (gray), and reversion/selected mutants (red). In *F*, SDs of independent triplicate experiments are shown as error bars with individual measurements shown as points. The k_cat_ error range for V2D9r is shown as a shaded bar.

We created designs using two versions of the PIP protocol, denoted versions 1 and 2 (see *Methods* and *SI Appendix*, *SI Methods* for details regarding differences in implementation of the PIP steps). In total, 33 to 39 and 29 to 30 residue positions were designed (allowed to change amino acid residue) in versions 1 and 2, respectively. We selected 32 designs for experimental testing, 14 from version 1 and 22 from version 2. Designs were named according to the version of PIP used to create them (V1 and V2), a design number (D1, D2, …), and an appended “r” to indicate whether any mutations were reverted to the wild-type residue based on visual inspection (for details, see *SI Appendix*, Tables 8 and 9 and Figs. 3 and 4). We chose designs that maximized the gap in Rosetta score between models that correctly place the catalytic residue (<1 Å restraint satisfaction, defined as the maximum distance between a restrained atom and its defined position) and models that do not correctly position the catalytic residue (>2 Å restraint satisfaction). We also chose designs that were predicted to have few buried unsatisfied hydrogen bond donors or acceptors and that did not have significant sequence and structural similarity to other selected designs. Selected designs contained between 12 (V2D6r, V2D9r) and 32 (V1D7) mutations. For PIP version 1, all selected designs expressed in the insoluble fraction after cell lysis and had to be purified from inclusion bodies, as is common with KSI mutants (35). Of the designs purified from inclusion bodies, half were soluble after refolding. We selected one design to characterize in further detail based on an initial screen of catalytic activity (*SI Appendix*, Table 10), V1D8r. For version 2, we obtained one design that expressed in the soluble fraction, V2D9r. Both designs V1D8r and V2D9r were stable after purification as assessed by circular dichroism spectroscopy (*SI Appendix*, Fig. 5).

### Functional Characterization of Designed KSI Variants.

Both designs V1D8r and V2D9r showed robustly measurable enzymatic activity when using 5 (10)-estrene-3,17-dione as a substrate (Fig. 3*C*), enhancing catalysis by four to five orders of magnitude when compared to the water-catalyzed isomerization of the similar 5-androstene-3,17-dione (36). To test for the ability of PIP to accurately position functional groups, we reverted the glutamate in the designs back to the original wild-type aspartate. Because of the sensitivity to functional group positioning observed in wild-type when adding a methylene group going from aspartate to glutamate and if we indeed correctly positioned the new glutamate in the design, we expected a considerable drop in catalytic activity in the design upon subtracting the methylene group again. As predicted by this model, for both designs V1D8r and V2D9r, we found a substantial reduction in k_cat_ in the E38D reversion mutant; the activities of both E38D mutants were near the detection limit of the assay and were reduced compared to the designs with E38 by at least 41-fold and 119-fold for V1D8r and V2D9r, respectively (Fig. 3*D*). This reduction was not simply due to loss of protein stability, as both E38D reversion mutants in the design background were folded (*SI Appendix*, Fig. 5 *B*–*D*). Notably, these fold changes are similar to the 103-fold change in k_cat_ between wild-type KSI and the D38E mutation (Fig. 3*D*; Table 1). Taken together, these results suggest that the designed backbone geometries successfully altered the enzyme’s preference for its catalytic residue. We note that the designs were overall less active than both wild-type and D38E KSI (Table 1). There are many potential reasons for this finding (*SI Appendix*, Note 4), including the observation that the designs are monomeric at the concentrations of the enzyme assay, whereas wild-type KSI functions as a dimer (*SI Appendix*, Fig. 5*E*). Additionally, our designs contain a large number of mutations (19 and 12 for V1D8r and V2D9r, respectively) that could affect active site electrostatics important for catalysis (37). Predicting the energetics of polar interactions making up protein functional sites with sufficient accuracy is a formidable problem, and we note that PIP (like other computational design methods) does not consider possible requirements of catalysis other than positioning (*Discussion*). However, our analysis suggests that the positioning of the catalytic residue’s carboxyl moiety, which PIP optimized for, is still an important determinant of catalytic activity. In particular, the design V2D9r has approximately the same fold reduction when changing glutamate to aspartate as wild-type KSI when changing aspartate to glutamate.

### Structural Characterization of Designed KSI Variants.

To assess whether V1D8r and V2D9r indeed adopted the designed backbone conformations, we determined crystal structures of the two designs. Both structures contained a ligand in the active site. For V1D8r, we observed density from deoxycholate retained from the purification process. V2D9r was cocrystallized with equilenin (which was present in the structure that was used as a basis for design) but also contained some residual density for deoxycholate (see next paragraph and *Methods*). For both designs, the electron density of the reshaped backbone region (residues 34 to 45 for V1D8r and 34 to 46 for V2D9r) was well resolved (*SI Appendix*, Fig. 6 *A* and *B*). Importantly, the backbone geometries of the reshaped backbone region in V1D8r and V2D9r were within 1.39 and 1.15 Å RMSD (N, C, Cα, and O backbone atoms) of the corresponding lowest-energy design models (Fig. 4*A*). For comparison, both the design structures and the computational models had conformations considerably different from the wild-type backbone (Fig. 4*A*). In the reshaped region, the design model of V1D8r and V2D9r differed from wild-type by 2.41 Å and 2.50 Å backbone RMSD, respectively. If considering the most variable segment (residues 37 to 42 for V1D8r and 37 to 43 for V2D9r), the design models for V1D8r and V2D9r differed from wild-type by 3.49 and 3.34 Å RMSD, respectively.

Next, we examined side-chain positioning, especially the catalytic glutamate carboxyl group. V1D8r and V2D9r (which was cocrystallized with equilenin) placed the catalytic carboxyl within 1.25 and 0.7 Å RMSD of the wild-type aspartate carboxyl, respectively (Fig. 4*B*). The overall heavy-atom RMSDs for buried designed residues in the reshaped segment [solvent-accessible surface area (SASA) of less than 40 Å^2^] were 1.43 and 1.07 Å for V1D8r and V2D9r, respectively (*SI Appendix*, Fig. 6 *C* and *D*). As noted above, the crystal structures of both designs showed at least partial occupancy of deoxycholate in the ligand-binding site, and it is conceivable that the bulky carboxyl moiety of the ligand changed the placement of the catalytic carboxyl group. This hypothesis is supported by the observation that the V2D9r crystal had partial occupancy of deoxycholate in three out of four asymmetric units, and the positioning of the catalytic residue in those monomers was significantly worse than in the asymmetric unit that contained only equilenin. In the asymmetric unit that contained only equilenin, we observed two distinct possibilities for the placement of the carboxyl of E38, which we modeled as alternate conformations (*SI Appendix*, Fig 6*E*). Despite the apparent flexibility of E38, the crystallographic data support the conclusion that the designed backbone is indeed capable of supporting the desired functional site geometry, as one of the alternate conformations is close to the wild-type carboxyl placement (0.75 Å restraint satisfaction, 0.67 Å carboxyl heavy-atom RMSD compared to the amide group of 38N of 1QJG; Fig. 4*C*). Taken together, the structural analysis shows that PIP can design novel backbone conformations that differ by >3 Å from their native counterparts with high accuracy, and the functional analysis demonstrates successful switching of the specificity in the designs from aspartate to glutamate.

Finally, we tested the robustness of V2D9r’s redesigned backbone segment to mutation. To determine whether the activity of V2D9r was dependent on any particular residue in the redesigned region (Fig. 4 *C*–*E*), we performed an experimental alanine scan along all mutated residues. We also made reversion mutants for residues whose backbone atoms did not move significantly between the wild-type and the design conformations, as well as a T39S mutant that we hypothesized might alleviate problems with buried polar groups. No mutation affected the k_cat_ more than twofold except for the catalytic glutamate (Fig. 4*F*; *SI Appendix*, Note 5), suggesting that the designed new backbone conformation depends on several interactions to adopt a catalytically competent conformation, as well as the glutamate as a general base.

## Discussion

We introduced and validated methods to accurately position amino acid functional groups in proteins by computational design in cases that require substantial alterations of the protein backbone (Fig. 1). We first developed and benchmarked two robotics-inspired sampling methods, FKIC and LHKIC, that generate and predict the structures of backbone conformations with high accuracy (Fig. 2). We then integrated these methods into a design protocol, PIP, to accurately position side-chain functional groups by remodeling the backbone (Fig. 1) and validated the approach experimentally by functional analysis and solving crystal structures of designs with reshaped backbone regions (Figs. 3 and 4).

FKIC leads to considerable improvements over the two approaches it combines, the fragment-independent loop modeling method NGK (26) and the fragment insertion–based prediction approach CCD (24) (Fig. 2*B*). In addition to subångstrom structure prediction accuracy, our results demonstrate that FKIC provides up to ∼140- fold improvement in sampling native-like conformations on the challenging problems of modeling local protein conformations with multiple segments and arbitrary secondary structure composition. This key advance in sampling efficiency paves the way to use FKIC in combination with LHKIC to design new local backbone geometries not seen in nature. Our results provide a proof of concept for such a design application.

We note that FKIC and LHKIC are conceived for generation, design, and prediction of new local backbone conformations and not for homology modeling that may require additional nonlocal changes in protein structure. Therefore, applications of FKIC to homology modeling of naturally occurring proteins may require integration of FKIC with more aggressive remodeling in the entire protein, not just a local region (*SI Appendix*, Note 6 and Table 11). It will be interesting in the future to test whether deep learning methods for protein structure predictions (14, 15) could be used to predict structures of designed sequences more rapidly than the robotics methods assessed here while also achieving subångstrom accuracy. To our knowledge, there are not yet systematic studies benchmarking the accuracy of deep learning methods on protein regions with irregular structures in the absence of multiple sequence alignments and structures of homologous proteins, as will be the case when designing conformations not seen in nature.

Despite the success with positioning a functional group that required reshaping of a sizeable backbone region, our results also highlight the considerable challenges faced when designing functional proteins. PIP in its current implementation optimizes positioning and is hence more suitable to designing specific geometries for binding rather than catalysis (which may require consideration of other determinants of catalysis not considered in current computational design methods and sometimes not even fully known). Moreover, certain functions may require switching between two or more approximately isoenergetic conformations. Such a scenario is much more challenging to engineer than optimizing for one deep energy minimum, which is sufficient for successful de novo design of protein structures. While the achieved carboxylate positioning in our designs is encouraging, it is not perfect, and accurately estimating the relative free energies of different alternative conformations in proteins is a current challenge common to all state-of-the-art atomistic modeling methods.

Kinetic analysis of V2D9r failed to reveal any specific residues that were key to stabilizing the catalytically competent loop conformation (Fig. 4; *SI Appendix*, Note 5), highlighting an important challenge in the design and modeling of precise local protein conformations: The energetic contributions to the stabilization of a particular backbone geometry may be distributed among many residues, which, combined with enormous sequence and conformational landscapes, makes it difficult to arrive at a minimum via successive single-residue substitution. Efficient sequence and conformational sampling are therefore crucial to the design of functional geometries. LHKIC addresses this challenge by pairing the structure search with sequence information from natural proteins, favoring local sequence/structure compatibility.

Naturally occurring proteins are often only marginally stable, so when reengineering them for new functions, it can be challenging to maintain stability. One approach to avoid this problem is to start with highly stable entirely de novo designed proteins into which one can build desired structural features (38). However, the idealized geometries of current de novo proteins may not be optimal for specific new functions such as molecular recognition. Here, FKIC/LHKIC and other methods (39) could provide a way to systematically reshape local regions to endow de novo designed proteins with new functions. The ability to sample both conformational and sequence space afforded by the robotics-inspired approaches and protocols presented here should help address current limitations and be useful in both the design and modeling of novel backbone conformations that enable specific functional geometries for binding or conformational switching (40) in de novo designed proteins.

## Methods

### Structure Prediction Simulations.

#### FKIC overview.

FKIC is based on the KIC protocol (17), but instead of sampling nonpivot φ/ψ torsions probabilistically from Ramachandran space, FKIC uses coupled φ/ψ/ω degrees of freedom from consecutive residues of protein fragments of size nine, three, or one to sample conformational space. During the low- and high-resolution sampling stages (*SI Appendix*, Fig. 1), each KIC move in the original KIC protocol is replaced by an FKIC move (Fig. 2*A*). An FKIC move consists of the following sequence of steps: 1) a fragment library (see the “Generation of fragment libraries” section in *SI Appendix*, *SI Methods*) is chosen at random from all available libraries (i.e., 9-mers, 3-mers, and 1-mers), 2) the chosen fragment library is searched for fragment alignment frames that (at least partially) overlap with the given target subsegment, 3) one of the alignment frames is chosen at random, 4) one of the 200 fragments contained in the given alignment frame is chosen at random, 5) the φ/ψ/ω torsions of that fragment are applied to the respective overlapping region of the given target subsegment, and 6) the segment is closed using KIC. Fragment libraries used for FKIC are the same as for the CCD protocol used in benchmark comparisons. Importantly, for benchmarking purposes, we ran simulations using fragment libraries that excluded homologs to the given query sequence (*SI Appendix*, *SI Methods*).

#### LHKIC overview.

LHKIC and FKIC share the same overall simulation protocol (Fig. 2; *SI Appendix*, Fig. 1). In LHKIC, the nonpivot φ/ψ/ω degrees of freedom are sampled from fragments picked by the loophash algorithm (20). At each KIC sampling step, we calculate the 6D transformation from the residue before the first pivot to the residue after the last pivot. We use the 6D transformation to query a precompiled loophash database (see “Generation of loophash databases” in *SI Appendix*, *SI Methods*). One 6D transformation query can return multiple loops. Torsions of a random loop from the returned loops are applied to the residues between the pivot residues.

#### Rosetta simulations.

FKIC and NGK benchmarking simulations were performed using the Rosetta macromolecular modeling and design suite (https://www.rosettacommons.org/software), revision 59052. The LHKIC method was developed later and used Rosetta revision 60022. KIC simulation results reported in Fig. 2*B* were taken from Ref. 26. The Rosetta “CCD” loop modeling method using fragment insertion and the cyclic coordinate descent closure technique (24) is described in Ref. 25. The NGK loop modeling method is described in Ref. 26. For FKIC simulations, NGK was modified to sample torsions from the generated fragment libraries. Similarly, for LHKIC, NGK was modified to sample torsions from loops picked using loophash (20). For control simulations that use native bond lengths and angles as input, we replaced the input structure with the native structure and disabled the randomization of torsions at the beginning of the simulation. Since the publication of the original KIC method, the Rosetta energy function has undergone several revisions, including the changes described in the “talaris2013” and “talaris2014” versions (41) and the latest improvements made in the “ref2015” version (21). The ref2015 energy function (21) was used for all benchmarks unless otherwise noted. Compared to the other energy functions, ref2015 showed a consistent performance improvement (*SI Appendix*, Table 12). For each test protein in each benchmark set (see below), we generated 500 models with FKIC and calculated the backbone heavy-atom RMSD of each target segment after aligning the protein without the modeled segment to its crystal structure. We also measured the median run time to determine whether any increased sampling performance increases computational cost (*SI Appendix*, Table 1A).

Full descriptions of RosettaScripts code and command lines can be found in *SI Appendix*, *SI Methods*.

#### Benchmark datasets.

The 12-residue “Standard” benchmark dataset was as previously described (17, 26, 42). The 16-residue “Mixed Segment” dataset consists of 30 structures from the PDB containing 16-residue target segments in which each segment has 5 to 11 residues that contain α-helices or β-strands. The 10-residue “Multiple Segments” dataset consists of 30 structures from the PDB, each containing a pair of 10-residue interacting target segments. We also constructed two analogous sets that contain either two eight-residue segments or two 12-residue segments (*SI Appendix*, Note 2 and Tables S6 and S7). More details on the benchmark datasets are in *SI Appendix*, *SI Methods*.

#### Preparation of benchmark input structures.

To exclude information on the native conformation of the target segment(s) for all benchmark datasets, all side chains in the segment(s) as well as side chains within 10 Å of the segment(s) (based on all-atom pairwise distance measurements) were removed. The backbone information was removed by changing the segment into an extended conformation with idealized bond lengths and angles. The datasets were constructed with an openly available script: https://github.com/Kortemme-Lab/benchmark_set_construct.

#### Fragment distance calculation in structure prediction.

The chord distance (43) was calculated between pairs of fragments. The chord distance between two angles is defined as *D*^2^(θ_1_, θ_2_) = 2 − 2cos(θ_1_ − θ_2_). In our case, this value was calculated for backbone dihedral angles and summed over paired residues between fragments and target loops: $\langle D\rangle =\frac{1}{n}\sum_{i}^{n}\left(\frac{1}{2}D^{2}\left(\phi_{1}^{i},\phi_{2}^{i}\right)+\frac{1}{2}D^{2}\left(\psi_{1}^{i},\psi_{2}^{i}\right)\right)$, with *n* = 3 defining 3-mer fragments. For example, 〈D〉 will have a minimum of 0 if the angles match exactly and a maximum of 4 if the angles differ by 180 degrees.

### PIP Design Protocol.

#### Overview.

We created designs using two versions of the PIP protocol, denoted versions 1 and 2, which differed in several details. Version 1 was developed before FKIC and LHKIC and therefore used NGK for both model generation (step 1) and structure prediction (step 3). In step 1, we varied the length of the remodeled active site region from 0 to −6 residues (relative to its native length). In subsequent steps, we made comparisons only between segments of the same length to avoid biases toward longer segments that can make more favorable interactions at the expense of loss of conformational entropy not considered in Rosetta. Sequence design (step 2) was done using fixed-backbone rotamer sampling. Residues within 4 Å of the active site backbone segment were designed (i.e., allowed to change amino acid identity) excluding residues Y14, F54, D99, A114, and F116 that are important for catalysis. In total, 33 to 39 residues were allowed to design, depending on segment length. Designs from step 2 to be evaluated in step 3 were selected with a probability proportional to their Boltzmann-weighted Rosetta total scores. This approach was intended to improve the diversity of the selected designs while still selecting more favorable (low-scoring) designs. The designs selected by this procedure for experimental testing either retained the native segment length or shortened the segment by one residue.

In version 2, we made several changes: We used LHKIC for model generation (step 1) and FKIC for structure prediction (step 3). This strategy takes advantage of the ability of LHKIC to sample both sequence and structure simultaneously in step 1 (as fragment picking in LHKIC is independent of the starting sequence). Conversely, FKIC is better suited to predicting conformations given a sequence in step 3, since FKIC picks fragments based on the input sequence. In step 2, we incorporated a small degree of backbone flexibility into the design process by using the Rosetta FastDesign method, which iterates fixed-backbone sequence design and fixed-sequence structure minimization. Because this design algorithm is more computationally expensive than that from version 1, we made fewer designs per backbone model (10 instead of 50). Based on the results from version 1, we only considered two segment lengths: the native length and a one-residue deletion. We also allowed a different (and smaller) set of residues to design: 25 to 26 residues in the active site segment and four residues in a small β-hairpin (residues 74 to 77 in the dimer partner) that make interchain contacts with the active site segment. To select designs for step 3, we incorporated knowledge from additional metrics besides Rosetta score and functional group positioning. We used metrics including the number of buried unsatisfied and oversaturated hydrogen bonds, a fragment quality filter, total SASA, and Rosetta’s foldability metric (*SI Appendix*, *SI Methods*). Because it is unclear a priori how to prioritize these metrics, we used Pareto fronts consisting of the above metrics to choose designs for computational structure prediction (*SI Appendix*, Fig. 7). We also selected more designs than in version 1 (up to 422 instead of 200) for structure prediction in early iterations of step 3, taking advantage of the fact that FKIC requires fewer simulations than NGK to make subångstrom predictions.

In comparison, both versions of PIP used similar robotics-inspired approaches to conformational sampling, but PIP version 2 placed an additional emphasis on fragment-based sampling using FKIC/LHKIC and analysis of fragment quality using Pareto fronts. Fragment quality measures how well designs conform to local sequence/structure relationships observed in naturally occurring proteins, and designs with a better fragment quality might be expected to be more stable (44). Attention to fragment quality as a design metric may have resulted in several beneficial characteristics in design V2D9r, which was both more soluble when expressed in *Escherichia coli* and had a higher apparent T_M_ (*SI Appendix*, Fig. 5) than design V1D8r. However, our design sample is small and further exploration of the impact of fragment-based design on design success would be interesting.

#### Rosetta version.

PIP was run using Rosetta commit 10b6f2f8e20d70757e6b510def2ddcbeef172538 (PIP version 1) or revision 60048 (PIP version 2). We used the latest available score function for each PIP version, which were talaris2013 for PIP version 1 or ref2015 for PIP version 2.

More details on the PIP protocol are in the *SI Appendix*, *SI Methods*.

### Experimental Characterization.

#### Cloning and purification.

The 14 designs chosen for experimental tests from PIP version 1 were ordered from GenScript precloned into the pET-21a expression vector. For PIP version 2 and for characterization of V1D8r and the wild-type protein, we used an expression vector using parts from the modular yeast cloning toolkit (45), which was similar to pET-21a except that the cloning resulted in a glycine-serine genetic scar at the carboxyl terminus. Full sequences of ordered designs and vectors can be found in *SI Appendix*, Data 1. Proteins were expressed in *E. coli* BL21(DE3) cells. Wild-type KSI and design V2D9r were purified essentially as described previously (35, 46) with minor differences (*SI Appendix*, *SI Methods*).

#### Activity assay.

Purified KSI variants were tested for catalytic activity using an absorbance assay. First, 5 (10)-estrene-3,17-dione was solubilized at 2.1 mM and serial diluted twofold down to 0.51 μM in 100% dimethyl sulfoxide. Then, 115 μL enzyme, prepared in 40 mM potassium phosphate, 2 mM dithiothreitol, and 1 mM ethylenediaminetetraacetic acid (pH 7.2), was then added to 5 μL of substrate for final substrate concentrations between 520 and 0.51 μM. The K_M_ and k_cat_ values for the wild-type enzyme and designs V1D8r and V2D9r were measured at enzyme concentrations between 0.5 and 18 μM. For reversion and alanine scan mutations, k_cat_ values were measured in triplicate at 512 μM substrate. Absorbance at 248 nm was tracked for 5 min at room temperature in a Varian Cary 50 Bio Ultraviolet-Visible spectrophotometer using a 1-cm pathlength. The first 30 to 60 s of each reaction were excluded to allow the reaction to reach steady state.

#### X-ray crystallography.

Designed proteins were crystallized in 1 M ammonium sulfate (design V1D8r) or 1.6 M ammonium sulfate, 50 mM potassium phosphate (pH 7.2) (design V2D9r) using the hanging drop method. For design V2D9r, an equal volume of 2 mM equilenin (CAS 517–09-9 from Steraloids Inc., catalog ID E0400-000) was added to each drop. For details on X-ray data collection and processing, see *SI Appendix*, *SI Methods*.

#### Structure determination.

We obtained initial phase information for calculation of electron density maps by molecular replacement using the program Phaser (47), as implemented in the PHENIX suite (48). For the V1D8r structure, we identified a single copy of the protein in the asymmetric unit using the coordinates from a previous KSI model, and for the V2D9r structure, we identified four copies of the protein in the asymmetric unit. Both solutions were consistent with an analysis of Matthews probabilities for the observed unit cell and molecular weight of the protein (49, 50).

We manually rebuilt the molecular replacement solutions using the resulting electron density maps, followed by iterative refinement of atomic positions, individual atomic displacement parameters (B-factors) with a translation–libration–screw rotation model, and occupancies, using riding hydrogen atoms and automatic weight optimization, until the model reached convergence. Throughout the course of manual model building, electron density corresponding to several ligand molecules became apparent, which we were able to model. In the V1D8r structure, we observed electron density for two steroid-like molecules, one occupying the KSI active site and a second nestled at a crystal contact. These densities were modeled using deoxycholate, which was present in one of the purification buffers used to prepare the crystallization samples. Additionally, we identified two phosphate ions in this structure. In the V2D9r structure, we also saw density for steroid ligands in the active sites of each of the four copies of the enzyme. In this case, the modeling was challenging, because the samples were exposed to both deoxycholate (during purification) and equilenin (postpurification), and electron density features suggested that there could be a mixture of both ligands represented in the electron density. We attempted to model various combinations of the ligands into the active site densities and found that the electron density features could best be described by modeling equilenin in one active site (chain B), deoxycholate in one active site (chain D), and a mixture of both ligands in the other two active sites (chains A and C). Our choice to model the ligand densities in this way is based on both reduction of refinement R-factors, as well as on overall flatness of residual Fo-Fc difference density maps around the modeled ligands. In the V2D9r structure, we also modeled 12 sulfate ions. All model building was performed using Coot (51), and refinement steps were performed with phenix.refine within the PHENIX suite (48, 51). Restraints for the ligands were calculated using phenix.elbow (52). Further information regarding model building and refinement is presented in *SI Appendix*, Table 13.

#### RMSD and SASA calculations.

For all RMSD calculations, structures were aligned to all residues except those involved in the RMSD calculation. To calculate backbone RMSDs that involved comparing the shorter V1D8r segment to the full-length wild-type protein, we had to exclude one residue in the wild-type structure. We chose to exclude residue 38, as this resulted in the lowest RMSD between the design and the wild-type protein. Per-residue SASA was calculated using the SasaCalc class in PyRosetta version 2021.12+release.ed6a5560506, which uses the LeGrand approximation of molecular surface area (53).

## Supplementary Material

Supplementary File

## Data Availability

Coordinates and structure files have been deposited to the PDB with accession codes 6UAD (V1D8r) and 6UAE (V2D9r). Rosetta source code is available from rosettacommons.org. PIP is available at https://github.com/Kortemme-Lab/pull_into_place. The parameter files used to design KSI are available at https://github.com/Kortemme-Lab/ksi_inputs. All study data are included in the article and/or *SI Appendix*.
