# Supplementary material for: Accurate positioning of functional residues with robotics-inspired computational protein design
Source: Proc Natl Acad Sci U S A. 2022 Mar 7;119(11):e2115480119. doi: 10.1073/pnas.2115480119 (PMC8931229; doi:10.1073/pnas.2115480119)
Supplement: Supplementary File [file pnas.2115480119.sapp.pdf]

SUPPLEMENTARY MATERIALS

Title:

Accurate positioning of functional residues with robotics-inspired computational protein design

Authors:
Cody Krivacic[1,2]*, Kale Kundert[2,3]*, Xingjie Pan[1,2]*, Roland A. Pache[2,7]*, Lin Liu[2], Shane O Conchúir[2], Jeliazko R. Jeliazkov[4], Jeffrey J. Gray[5], Michael C. Thompson[2,8], James S. Fraser[1,2,3,6], Tanja Kortemme[1,2,3,6$].

*These authors contributed equally to this work

Supplementary Methods
Supplementary Notes 1 – 6
Supplementary Tables 1 – 14
Supplementary Figures 1 – 7
References Cited in the Supplementary Materials

SUPPLEMENTARY METHODS

GENERATION AND PREDICTION OF NEW CONFORMATIONS (FKIC AND LHKIC)

Rosetta scripts & command line for CCD

We used the following Rosetta scripts to run the CCD benchmark simulations:

```
<ROSETTASCRIPTS>
  <TASKOPERATIONS>
    <RestrictToLoops name="loop" loops_file="%%loop_file%%"/>
  </TASKOPERATIONS>
  <MOVERS>
    <LoopmodelWrapper name="modeler" loops_file="%%loop_file%%" fast="%%fast%%"/>
  </MOVERS>
  <PROTOCOLS>
    <Add mover_name=”modeler”/>
  </PROTOCOLS>
</ROSETTASCRIPTS>
```

Let the home directory of Rosetta be */path/to/rosetta/main*; the input structure be *my_structure.pdb*; the loop file be *my_structure.loop*; the fragment files be *my_structure.200.9mers.gz* and *my_structure.200.3mers.gz*; and the Rosetta script XML file be *loopmodel.xml*. Then the command to run one simulation is:

/path/to/rosetta/main/source/bin/rosetta_scripts.linuxgccrelease -database /path/to/rosetta/main/database -in:file:s my_structure.pdb -parser:protocol loopmodel.xml -parser:script_vars loop_file=my_structure.loop fast=no -out:prefix prefix -overwrite -loops:remodel quick_ccd -loops:refine refine_ccd -ex1 -ex2 -loops:frag_sizes 9 3 1 -loops:frag_files my_structure.200.9mers.gz my_structure.200.3mers.gz

Rosetta scripts & command line for NGK

We used the following Rosetta script to run the NGK benchmark simulations:

```
<ROSETTASCRIPTS>
  <TASKOPERATIONS>
    <RestrictToLoops name="loop" loops_file="%%loop_file%%"/>
  </TASKOPERATIONS>
  <MOVERS>
    <LoopModeler name="modeler" config="kic" loops_file="%%loop_file%%" fast="%%fast%%" />
  </MOVERS>
```

```
    <PROTOCOLS>
        <Add mover_name=”modeler”/>
    </PROTOCOLS>
</ROSETTASCRIPTS>
```

Let the home directory of Rosetta be */path/to/rosetta/main*; the input structure be *my_structure.pdb*; the loop file be *my_structure.loop*; and the Rosetta script XML file be *loopmodel.xml.* Then the command line to run one simulation is:

/path/to/rosetta/main/source/bin/rosetta_scripts.mysql.linuxgccrelease -database /path/to/rosetta/main/main/database -in:file:s my_structure.pdb -parser:protocol loopmodel.xml -parser:script_vars loop_file=my_structure.loop fast=no -out:prefix prefix -overwrite

Rosetta scripts & command line for FKIC

We used the following Rosetta script to run FKIC benchmark simulations:

```
<ROSETTASCRIPTS>
    <TASKOPERATIONS>
        <RestrictToLoops name="loop" loops_file="%%loop_file%%"/>
    </TASKOPERATIONS>
    <MOVERS>
        <LoopModeler
            name="modeler"
            config="kic_with_frags"
            loops_file="%%loop_file%%"
            fast="%%fast%%">
        </LoopModeler>
    </MOVERS>
    <PROTOCOLS>
        <Add mover_name=”modeler”/>
    </PROTOCOLS>
</ROSETTASCRIPTS>
```

Let the home directory of Rosetta be */path/to/rosetta/main*; the input structure be *my_structure.pdb*; the loop file be *my_structure.loop*; the fragment files be *my_structure.200.9mers.gz* and *my_structure.200.3mers.gz*; and the Rosetta script XML file be *loopmodel.xml.* Then the command line for one simulation is:

/path/to/rosetta/main/source/bin/rosetta_scripts.mysql.linuxgccrelease -database /path/to/rosetta/main/database -in:file:s my_structure.pdb -parser:protocol loopmodel.xml -parser:script_vars loop_file=my_structure.loop fast=no -out:prefix prefix -overwrite -loops:frag_sizes 9 3 1 -loops:frag_files my_structure.200.9mers.gz my_structure.200.3mers.gz

Generation of loophash databases

We generated the loophash databases using the *loophash_createfiltereddb* application and the VALL database distributed with Rosetta. The command line is:

mpirun -np 32 loophash_createfiltereddb.mpi.linuxgccrelease -lh:db_path loophash_db/ -in:file:vall path_to_rosetta_tools_repository/tools/fragment_tools/vall.jul19.2011.gz -lh:loopsizes 3 4 5 6 7 8 9 10 11 12 13 14 -lh:num_partitions 32 -lh:createdb_rms_cutoff 4.5 6 7.5 9 10.5 12 13.5 15 16.5 18 19.5 21

where *loophash_db* is the output path.

Rosetta scripts & command line for LHKIC

We used the following Rosetta script to run the LHKIC benchmark simulations:

```
<ROSETTASCRIPTS>
   <MOVERS>
      <LoopModeler name="modeler" config="loophash_kic" loops_file="%%loop_file%%"
fast="%%fast%%" />
   </MOVERS>
   <PROTOCOLS>
      <Add mover_name="modeler"/>
   </PROTOCOLS>
</ROSETTASCRIPTS>
```

Let the home directory of Rosetta be */path/to/rosetta/main*; the input structure be my_structure.pdb; the loop file be *my_structure.loop*; the Rosetta script XML file be loopmodel.xml; and the path to the loophash database be *path_to_loophash_db*. Then the command line to run one simulation is:

/path/to/rosetta/main/source/bin/rosetta_scripts.mysql.linuxgccrelease -database /path/to/rosetta/main/main/database -in:file:s my_structure.pdb -parser:protocol loopmodel.xml -parser:script_vars loop_file=my_structure.loop fast=no -out:prefix prefix -overwrite -lh:loopsizes 6 8 10 -lh:db_path path_to_loophash_db

By default, LHKIC does not mutate the sequence of the loop. When the *loophash_perturb_sequence* option is set to true, LHKIC applies the sequence of the returned loop to the pivot residues and the residues between the pivots.

Rosetta scripts & command line for simulations with native bond lengths and angles

For control simulations that use native bond lengths and angles as input, we replaced the Rosetta script XML file with the following:

```
<ROSETTASCRIPTS>
  <TASKOPERATIONS>
    <RestrictToLoops name="loop" loops_file="%%loop_file%%"/>
  </TASKOPERATIONS>
  <MOVERS>
    <LoopModeler
      name="modeler"
      config="kic_with_frags"
      loops_file="%%loop_file%%"
      fast="%%fast%%">
      <Build skip="True" />
    </LoopModeler>
  </MOVERS>
  <PROTOCOLS>
    <Add mover_name="modeler"/>
  </PROTOCOLS>
</ROSETTASCRIPTS>
```

12-Residue "*Standard*" benchmark dataset

The "Standard" set is a 12-residue loop benchmark dataset used in previous work[1-3]. This benchmark dataset consists of 45 protein structures from the PDB containing non-redundant 12-residue target segments without regular secondary structure, curated from two previously described datasets[4,5]. We used this dataset even though it is not ideal (for example, the conformation of several segments might be influenced by crystal contacts, see **Supplementary Note 1** and **Supplementary Table 5**) to facilitate comparison of FKIC with previous protocols. For each loop, we retained the N and Cα atoms of the N-terminal residue, as well as the Cα, C and O atoms of the C-terminal residue, which serve as loop anchor points for kinematic closure (as in ref.[1]).

16-Residue "Mixed Segment" benchmark dataset

The *Mixed Segment* benchmark dataset consists of 30 structures from the PDB containing 16-residue target segments. The target segments were derived from structures in the *Standard* benchmark dataset above using the following criteria:

- The crystallographic resolution of the experimentally determined structure is equal to or better than 2Å.
- Each segment has 5 to 11 residues that contain alpha helices or beta strands defined using DSSP[6] and the remainder of the segment is designated as loop.

- Residues in the segment are at least 4Å away from any chains or copies of the molecule in other asymmetric units, to avoid crystal contacts.
- The segment is at least 5 residues away from the chain termini.

The segment that satisfied all criteria with the lowest distance from the protein surface was selected.

10-Residue "*Multiple Segments*" benchmark dataset

The *Multiple Segments* benchmark dataset consists of 30 structures from the PDB each containing a pair of 10-residue interacting target segments. Structures were derived from the top8000 dataset[7] using the following criteria:

- The crystallographic resolution of the experimentally determined structure is equal to or better than 2Å.
- Each segment has less than 3 residues that have regular secondary structure, defined as above.
- Residues in the segment are at least 4Å away from any chains or copies of the molecule in other asymmetric units, to avoid crystal contacts.
- Each segment is at least 5 residues away from the chain termini.
- Segments in each pair are within 4Å and are separated by at least 5 residues in primary sequence.

The pair of segments that satisfied all criteria with the lowest distance from the protein surface was selected. We also constructed two analogous sets that contain either two 8-residue segments or two 12-residue segments (**Supplementary Note 2; Supplementary Table 6** and **Supplementary Table 7**).

Generation of fragment libraries

We generated libraries of 9-mer and 3-mer fragments for all benchmark cases using the fragment picking method described in ref.[8] (9-and 3-mers are established fragment sizes tested in a variety of Rosetta applications). The method selects fragments from a representative database of 16,801 protein chains extracted from the PDB and culled such that any two chains have at most 60% sequence identity. The fragment database is part of the Rosetta software and located in rosetta/tools/fragment_tools/vall.jul19.2011.gz. The fragments selected for each segment sequence position span a 3- or 9-residue frame, which overlaps with neighboring frames. Moreover, we allow sampling of 1-mer fragments, which consist of a single triplet of φ/ψ/ω torsions that are generated on the fly by the respective modeling protocol (see below) based on the 3-mer fragment library for the given position. The make_fragments.pl script in the rosetta/tools/fragment_tools/ directory integrates several data sources to maximize fragment quality, including sequence similarity and the detection of homologs using PsiBLAST[9], predicted secondary structure similarity using PsiPred[10] and prediction of preferred φ/ψ torsions and solvent accessibility using SPARKS-X[11]. Importantly, for benchmarking purposes, we ran simulations using fragment libraries that excluded homologs to the given query sequence, by providing the –nohoms flag to the make_fragments.pl script, which excluded all protein chains with a PsiBLAST E-value < 0.05[9] from the fragment picking process.

COMPUTATIONAL DESIGN OF NEW CONFORMATIONS (PIP PROTOCOL)

Overview

Input files. KSI designs were based on PDB structure 1QJG[12]. KSI is an obligate dimer, so we included both monomers in our initial structure. To design different loop lengths, we created several versions of the initial structure: one for each deletion of up to 6 residues, and one with wild-type length. We replaced N38 with a glutamate and relaxed the resulting models 100 times in the talaris2013 (version 1) or ref2015 (version 2) score function using FastRelax, with all atom coordinates restrained to their starting positions. The best (lowest-scoring) relaxed structure was then repacked 100 times, and the lowest-scoring model was used as a template for design.

The desired position of the E38 sidechain was defined in a restraint file (see below). Each atom in the E38 carboxylate group was restrained to the position of the corresponding atom in the N38 amide group in the starting structure (1QJG). We visually confirmed that N38 in 1QJG had the same rotameric conformation as D38 in wild-type KSI (PDB structure 8CHO). When working with the version 1 designs, we noticed that F54 sometimes changed its rotamer conformation, causing subsequent designed mutations to stabilize the altered conformation. For version 2, we addressed this issue by placing restraints on the zeta-carbon of F54 in a manner similar to the catalytic residue.

The residues being remodeled were defined in loop files (see below). We chose which residues to remodel based on proximity to secondary structure elements and intuition. Our goals were (i) to allow sufficient remodeling on either side of E38 to stabilize its new conformation, (ii) to anchor the loop in secondary structural elements, and (iii) to minimize loop length. With these considerations in mind, we remodeled segments that were 7-13 (version 1) or 12-13 (version 2) residues long. (While in principle other segment lengths could be tried, 12-13 residue loops resulted in the best designs from round 1, and allowed us to limit the considerable computational expense exploring large ensemble of potential conformations and sequences at each length). For version 2, we also remodeled a second 4-residue loop on the dimer interface (residues 199-202 using Rosetta numbering, residues 74-77 on chain B using PDB numbering), hoping to maintain favorable contacts between those residues and the catalytic loop.

The residues that were allowed to design (change amino acid identity) and repack (only change rotamer conformation) were specified in a resfile (see below). For version 1, any residue that had a sidechain atom within 4Å or 6Å of any loop atom in any model generated in PIP step 1 was allowed to design or repack, respectively. For version 2, we only allowed Rosetta to design residues on the catalytic loop, as well as four residues on the short dimerization loop which directly interacts with the catalytic loop (described above). Repackable residues were selected using the Rosetta clash-based repack shell selector. F54, A114, and F116 were not allowed to design in either version because they are known to be important for positioning the catalytic residue[13]. For version 1, each designed residue was allowed to change to any of the 20 canonical amino acids except cysteine (due to the potential for disulfide bonds) and histidine (due to the potential for pH-dependent behavior). For version 2, we used the LayerDesign task operation in Rosetta to determine which residue identities were allowed at each position. Since version 2 introduced backbone degrees of freedom during the design step, we specified a fold tree to keep conformational changes as local as possible (see below).

PIP Step 1: Build Models. We created models positioning the E38 carboxylate group by running approximately 20,000 NGK[2] (version 1) or LHKIC (version 2) simulations with restraints as described above. Backbone remodeling was limited to the loop defined in the appropriate loop file and design was allowed according to the appropriate resfile (see below). In version 1 of PIP, the initial coordinates of the loop being remodeled were discarded and rebuilt from scratch. This step was skipped for version 2. Only models that put all three restrained atoms within 0.6 Å (version 1) or 0.7 Å (version 2) of their intended positions were carried on to the next step.

PIP Step 2: Design Models. To stabilize models that correctly positioned E38, we ran 50 fixed-backbone (version 1) or 10 FastDesign (version 2) simulations per model, or more if there were relatively few models. Design was allowed according to the appropriate resfile (see below). For version 1, we picked 200 designs for PIP step 3 with probability proportional to their Boltzmann-weighted talaris2013 scores (in Rosetta energy units, REU). For version 2, we used a combination of Pareto fronts and thresholds to pick designs so that we could supplement information from the Rosetta score function with additional metrics. The exact parameters used to pick designs are included in a “picks” file, described below. The metrics for which we applied thresholds were the E38 restraint distance (defined as the maximum distance of the restrained atoms in E38 to their ideal position), the number of hydrogen bonds to the E38 sidechain, and the number of oversaturated hydrogen bonds. Models that passed these thresholds were included in the Pareto front calculation, whose metrics consisted of the total solvent-accessible surface area of the model, two “foldability” metrics that perform 60 brief forward-folding simulations on pieces of the loop and report the fraction of results that placed the segment’s N-terminus within 4 Å of the concomitant residue in the design structure, the E38 restraint distance, a fragment quality metric (see below), and the Rosetta fa_attr score. The Foldability metrics remove a portion of the design’s backbone, then rebuild it starting from the N-terminus of the deleted segment using fragment-based assembly. This is repeated 100 times, and the average distance of the C-terminus of the rebuilt segment to its position in the design is reported.

PIP Step 3: Structure Prediction. We computationally assessed our designs by running between 100 and 500 NGK (version 1) or FKIC (version 2) structure prediction simulations for each design; for version 2, we opted to perform fewer structure prediction simulations on a larger number of designs during the early rounds of design. Backbone movement was limited to the loop defined in the appropriate loop file (see below). The initial coordinates for that loop were discarded and rebuilt from scratch. Any design for which the lowest scoring decoy put all three carboxylate atoms within 1.2 Å of their intended positions was carried on to the design selection step. Furthermore, any decoy (regardless of score) that put all three carboxylate atoms within 0.6 Å of their intended positions was used as input for a second round of design simulations.

Design Selection. We picked designs to experimentally test by comparing quality metrics and visually inspecting models. The quality metrics are described in **Supplementary Table 8**. We paid particular attention to the score gap, which measures the difference in the score between the lowest-scoring model with under 1 Å restraint satisfaction and the lowest-scoring model with over 2 Å restraint satisfaction. Several designs were selected despite having a score gap of 0 REU, as they had multiple low-energy conformations. We also made an effort to pick designs from different sequence and structure clusters. Design sequences were clustered hierarchically such that inter-cluster distance

was no greater than the mean sequence distance (calculated according to the BLOSUM80 substitution matrix) across all designs. Structure clusters were formed hierarchically such that the RMSD between any two designs in the same cluster was no greater than 1.2 Å. We visually inspected the lowest scoring model for each design to eliminate those with irregular backbone or strained sidechain conformations.

Wildtype Reversions. For each design from PIP version 1 selected for experimental validation, we reran the structure prediction simulations (PIP Step 3) for each single wildtype reversion mutation. We then combined any reversions that had no apparent detrimental effect on our quality metrics and again ran the structure prediction simulations. In cases where the combination of all the individually acceptable reversions had a deleterious effect, we selected more conservative combinations of reversions for additional structure prediction simulations. If no acceptable combination of reversions could be found, no reversions were made (**Supplementary Table 8**). For PIP version 2, positions where the backbone was in a similar position to wildtype were reverted, or in some cases mutated to a residue picked by visual inspection (**Supplementary Table 9**), and designs with and without those reversions were ordered for experimental validation.

Input files for PIP

E38 constraints definition
We used the following energetic constraints file for PIP Version 1 and Version 2:

```
CoordinateConstraint CG  38 CA 1 12.159  64.031 -28.170 HARMONIC 0.0 1.0
CoordinateConstraint OE1 38 CA 1 10.881  63.345 -30.090 HARMONIC 0.0 1.0
CoordinateConstraint OE2 38 CA 1 11.759  65.409 -30.106 HARMONIC 0.0 1.0
```

Additional constraint on F54 for PIP version 2
For PIP Version 2, we included the following additional constraint to prevent a conformational change in the sidechain of F54 that was frequently observed in PIP Version 1:

```
CoordinateConstraint CZ  54 CA 1 15.196  64.334 -29.952 HARMONIC 0.0 1.0
```

Loop definitions for design V1D8r
We used the following loop definition for PIP Version 1 models with a single deletion in the loop region:

```
LOOP 34 45 45 0 1
```

The loop definition for input models with no deletions included residue 46.

Loop definitions for design V2D9r
For PIP Version 2, we used the following loop definitions:

```
LOOP  26  51  40 0 0
```

LOOP 198 203 200 0 0

<u>Design step resfile for PIP Version 1</u>
We used the following resfile for PIP Version 1 (for input models with a single deletion):

```
NATRO
START
# Design residues in the loop itself. Don't move the catalytic residue,
# because we want to find designs which stabilize that rotamer.
34 - 37 A NOTAA HC
38 A NATRO
39 - 45 A NOTAA HC
# Design any residue that has a sidechain atom within 4A of any loop atom in
# any input model. Phe53, Ala113, and Phe115 are excluded because they are
# known to be important for positioning the catalytic residue.
29 A NOTAA HC
30 A NOTAA HC
31 A NOTAA HC
32 A NOTAA HC
33 A NOTAA HC
46 A NOTAA HC
48 A NOTAA HC
49 A NOTAA HC
50 A NOTAA HC
52 A NOTAA HC
54 A NOTAA HC
56 A NOTAA HC
57 A NOTAA HC
108 A NOTAA HC
109 A NOTAA HC
110 A NOTAA HC
111 A NOTAA HC
112 A NOTAA HC
114 A NOTAA HC
116 A NOTAA HC
117 A NOTAA HC
120 A NOTAA HC
198 B NOTAA HC
199 B NOTAA HC
200 B NOTAA HC
201 B NOTAA HC
# Repack any residue that has a sidechain atom within 6A of any loop atom in
# any input model.
10 A NATAA
11 A NATAA
13 A NATAA
14 A NATAA
15 A NATAA
16 A NATAA
17 A NATAA
```

18 A NATAA
23 A NATAA
26 A NATAA
27 A NATAA
28 A NATAA
47 A NATAA
51 A NATAA
53 A NATAA
55 A NATAA
58 A NATAA
59 A NATAA
60 A NATAA
62 A NATAA

For inputs with no deletions, we included the additional loop residue with the tag NOTAA HC and adjusted the residue numbers of post-loop positions accordingly.

Design step resfile for PIP Version 2

We used the following resfile for PIP version 2:

```
NATRO
START
34      A NOTAA CH
35      A NOTAA CH
36      A NOTAA CH
37      A NOTAA CH
38      A PIKAA E
39      A NOTAA CH
40      A NOTAA CH
41      A NOTAA CH
42      A NOTAA CH
43      A NOTAA CH
44      A NOTAA CH
45      A NOTAA CH
46      A NOTAA CH
199     B NOTAA CH
200     B NOTAA CH
201     B NOTAA CH
202     B NOTAA CH

# Repack positions
# ================
# The following repack positions were chosen by the clash-based repack
# shell creator (excluding the ligand).

14      A NATAA
```

```
30     A NATAA
50     A NATAA
51     A NATAA
54     A NATAA
55     A NATAA
95     A NATAA
109    A NATAA
111    A NATAA
112    A NATAA
113    A NATAA
114    A NATAA
115    A NATAA
116    A NATAA
121    A NATAA
127    B NATAA
204    B NATAA
225    B NATAA
227    B NATAA

# The following repack positions were added after visual inspection of
# clash-based repack shell.

10     A NATAA
13     A NATAA
17     A NATAA
25     A NATAA
52     A NATAA
53     A NATAA
56     A NATAA
57     A NATAA
58     A NATAA
108    A NATAA
110    A NATAA
117    A NATAA
118    A NATAA
126    B NATAA
128    B NATAA
228    B NATAA
```

<u>Picks file</u>

Thresholds and Pareto front metrics for PIP version 2 were defined using a YAML-formatted file. We used the following YAML-formatted file to define the metrics to be used as thresholds and in Pareto fronts for picking designs for step 3 in PIP Version 2. Designs which meet the criteria defined under “threshold” are passed into the Pareto front. Metrics listed under “pareto” are used to define the

Pareto front. The “depth” value describes how many times the Pareto front is applied, and the “epsilon” value defines how close two points can be in all Pareto dimensions being considered before they are considered to be the same and one is excluded. Its units are the percent of the range of points between the 10th and 90th percentiles. Let *restraint_dist_e38* be the E38 restraint distance, *h_bonds_to_e38_sidechain* be the number of hydrogen bonds to E38, *oversaturated_h_bonds* be the number of oversaturated hydrogen bonds, *fa_attr* be the attractive Van der Waals term in the Rosetta energy function, *max_9_residue_fragment_rmsd_c_alpha* be the Fragment Quality score (see below), *total_sasa* be the total solvent-accessible surface area, *foldability_37_44* be the Foldability score (described above) for residues 37 through 44, and *foldability_35_41* be the Foldability score for residues 35 through 41.

threshold:
- restraint_dist_e38 < 0.8
- h_bonds_to_e38_sidechain == 0
- oversaturated_h_bonds == 0

pareto:
- fa_attr
- restraint_dist_e38
- max_9_residue_fragment_rmsd_c_alpha
- total_sasa
- foldability_37_44
- foldability_35_41

depth: 1
epsilon: 0.5

Fragment Quality Analysis for Designed Regions

The fragment quality metric assesses the geometric similarity between 9-residue fragments in the designed segments and fragments of natural proteins in the PDB, as described in ref.[14]. Specifically, we picked protein fragments from the PDB based on their similarity in sequence, predicted solvent exposure, and predicted secondary structure to the post-simulation design sequence and determined the RMSD of the backbone atoms in each fragment to the final structure. We then determined the lowest RMSD at each position being evaluated (assigning the RMSD to position at the center of each nine residue fragment). The fragment score filter uses the highest value of these lowest fragment RMSDs at each position.

PIP Step 1: Build Models

We used the following command line for the Build Models step in PIP Version 1. Let the “main” directory in Rosetta be */path/to/rosetta/main/*; the (relaxed) input PDB be $INPUT_PDB; the

(unrelaxed) native PDB be $NATIVE_PDB; the full-atom and centroid scorefunction parameters for the talaris2013 scorefunction be "EQU.fa.params" and "EQU.cen.params", respectively; the resfile be $RESFILE; the constriants file be $CONSTRAINTS and the loops file be $LOOP.

```
/path/to/rosetta/main/source/bin/loopmodel.linuxgccrelease \
-in:file:s $INPUT_PDB \
-in:file:native $NATIVE_PDB \
-in:file:extra_res_fa "EQU.fa.params" \
-in:file:extra_res_cen "EQU.cen.params" \
-in:file:fullatom \
-out:overwrite \
-out:pdb_gz \
-packing:ex1 \
-packing:ex2 \
-packing:extrachi_cutoff 0 \
-packing:resfile $RESFILE \
-constraints:cst_fa_weight 1.0 \
-constraints:cst_fa_file $CONSTRAINTS \
-loops:loop_file $LOOP \
-loops:remodel "perturb_kic" \
-loops:refine "refine_kic" \
-loops:kic_rama2b \
-loops:kic_omega_sampling \
-loops:allow_omega_move "true" \
-loops:ramp_fa_rep \
-loops:ramp_rama \
```

For PIP Version 2, we used the following Rosetta script to build new backbone geometries. Let the loops file be $LOOPS_PATH. Definitions common to all steps are found in "shared_defs.xml", described below. For all Rosetta scripts, variables are filled in by the PIP package.

```
<ROSETTASCRIPTS>

  {% include "shared_defs.xml" %}

  <TASKOPERATIONS>
    <RestrictToRepacking name="repackonly"/>
  </TASKOPERATIONS>

  <MOVERS>
    <LoopModeler name="modeler"
      config="loophash_kic"
      scorefxn_fa="scorefxn_cst"
      task_operations="resfile,repackonly,ex,aro,curr"
      loops_file="$LOOPS_PATH"
      loophash_perturb_sequence="yes"
```

```
      loophash_seqposes_no_mutate="38"
      fast="no"
    />
  </MOVERS>

  <PROTOCOLS>
    <!-- Constraints read from command line -->
    <Add mover_name="modeler"/>
    <Add mover_name="writer"/>
  </PROTOCOLS>

  <OUTPUT scorefxn="scorefxn"/>

</ROSETTASCRIPTS>
```

The PIP package also builds the command line, but a representative example is shown below. Let the path to the “main” folder in Rosetta be */path/to/rosetta/main/*; the (relaxed) input PDB be $INPUT_PDB; the (unrelaxed) native PDB be $NATIVE_PDB; the folder where models are to be saved be $OUPUT_FOLDER; the name of the particular design be $OUTPUT_NAME; the “build models” Rosetta script be *build_models.xml*; the resfile path be $RESFILE_PATH; the path to the constraints file be $CONSTRAINTS, and the path to the loophash database be *path_to_loophash_db*.

```
/path/to/rosetta/main/source/bin/rosetta_scripts.linuxgccrelease \
-database /path/to/rosetta/main/database/ \
-in:file:s $INPUT_PDB \
-in:file:native $NATIVE_PDB \
-out:prefix $OUTPUT_FOLDER \
-out:suffix $OUTPUT_NAME \
-out:no_nstruct_label -out:overwrite -out:pdb_gz \
-out:mute protocols.loops.loops_main \
-parser:protocol build_models.xml \
-packing:resfile $RESFILE_PATH \
-constraints:cst_fa_file $CONSTRAINTS \
-lh:loopsizes 6 7 8 9 10 11 12 13 14 \
-lh:db_path path_to_loophash_db
```

PIP Step 2: Design Models

We used the following command line to design models in step 2 of PIP Version 1. Let the “main” directory in Rosetta be */path/to/rosetta/main/*; the (relaxed) input PDB be $INPUT_PDB; the full-atom

and centroid scorefunction parameters be “EQU.fa.params” and “EQU.cen.params”, respectively, and the resfile be $RESFILE.

```
/path/to/rosetta/main/source/bin/fixbb.linuxgccrelease \
-in:file:s $INPUT_PDB \
-in:file:extra_res_fa "EQU.fa.params" \
-in:file:extra_res_cen "EQU.cen.params" \
-out:overwrite \
-out:pdb_gz \
-packing:ex1 \
-packing:ex2 \
-packing:extrachi_cutoff 0 \
-packing:use_input_sc \
-packing:resfile $RESFILE \
```

For PIP Version 2, we defined a custom fold tree for the design step:

```
FOLD_TREE
EDGE   1  39 -1
EDGE 100   1  3
EDGE 100  40 -1
EDGE 100 125 -1
EDGE 100 225  1
EDGE 100 251  2
EDGE 126 200 -1
EDGE 225 126  4
EDGE 225 201 -1
EDGE 225 250 -1
```

We then used the following Rosetta script to design sequences for the new backbone geometries. Let the path to the fold tree file be $FOLDTREE. Definitions common to all steps are found in “shared_defs.xml”, described below.

```
<ROSETTASCRIPTS>

  {% include "shared_defs.xml" %}

  <RESIDUE_SELECTORS>
    <Index name="turn" resnums="200-201"/>
  </RESIDUE_SELECTORS>

  <TASKOPERATIONS>
    <LayerDesign name="layer"
        ignore_pikaa_natro="yes"/>
```

```
  <ConsensusLoopDesign name="abego"
      residue_selector="turn"
      include_adjacent_residues="no"/>
 </TASKOPERATIONS>

 <MOVERS>
  <AtomTree name="foldtree" fold_tree_file="$FOLDTREE"/>
  <AtomTree name="unfoldtree" simple_ft="yes"/>
  <AddChainBreak name="break_loop" resnum="39" change_foldtree="no"/>
  <AddChainBreak name="break_turn" resnum="200" change_foldtree="no"/>
  <FastDesign name="fastdesign"
      task_operations="resfile,layer,abego,ex,aro,curr"
      scorefxn="scorefxn_cst" >
    <MoveMap bb="no" chi="yes" jump="no">
      <Span begin="26"  end="51"  chi="yes" bb="yes"/>
      <Span begin="198" end="203" chi="yes" bb="yes"/>
    </MoveMap>
  </FastDesign>
 </MOVERS>

 <PROTOCOLS>
  <Add mover_name="nativebonus"/>
  <Add mover_name="cst"/> <!-- Added via mover b/c command-line ignored. -->
  <Add mover_name="foldtree"/>
  <Add mover_name="break_loop"/>
  <Add mover_name="break_turn"/>
  <Add mover_name="fastdesign"/>
  <Add mover_name="unfoldtree"/> <!-- Otherwise Foldability segfaults. -->
  <Add mover_name="writer"/>
 </PROTOCOLS>

 <OUTPUT scorefxn="scorefxn"/>

</ROSETTASCRIPTS>
```

A representative command line is shown below. Let the path to the “main” folder in Rosetta be */path/to/rosetta/main/*; the (relaxed) input PDB be $INPUT_PDB; the (unrelaxed) native PDB be $NATIVE_PDB; the folder where models are to be saved be $OUPUT_FOLDER; the name of the particular design be $OUTPUT_NAME; the “design models” Rosetta script be *design_models.xml*, and the resfile path be $RESFILE_PATH.

```
/path/to/rosetta/main/source/bin/rosetta_scripts.linuxgccrelease \
-database /path/to/rosetta/main/database/ \
-in:file:s $INPUT_PDB \
```

```
-in:file:native $NATIVE_PDB \
-out:prefix $OUTPUT_FOLDER \
-out:suffix $OUTPUT_NAME \
-out:no_nstruct_label -out:overwrite -out:pdb_gz \
-out:mute protocols.loops.loops_main \
-parser:protocol design_models.xml \
-packing:resfile $RESFILE_PATH \
```

PIP Step 3: Structure Prediction

We used the following command line to design models in step 2 of PIP Version 1. Let the “main” directory in Rosetta be */path/to/rosetta/main/*; the (relaxed) input PDB be $INPUT_PDB; the full-atom and centroid scorefunction parameters be “EQU.fa.params” and “EQU.cen.params”, respectively, and the loops file be $LOOPS.

```
/path/to/rosetta/main/source/bin/loopmodel.linuxgccrelease \
-in:file:s $INPUT_PDB \
-in:file:native $NATIVE_PDB \
-in:file:extra_res_fa "EQU.fa.params" \
-in:file:extra_res_cen "EQU.cen.params" \
-in:file:fullatom \
-out:pdb_gz \
-out:overwrite \
-packing:ex1 \
-packing:ex2 \
-packing:extrachi_cutoff 0 \
-loops:loop_file $LOOPS \
-loops:remodel "perturb_kic" \
-loops:refine "refine_kic" \
-loops:kic_rama2b \
-loops:kic_omega_sampling \
-loops:ramp_fa_rep \
-loops:ramp_rama \
```

For PIP Version 2, we used the following Rosetta script to predict the structures of picked designs. Let the loops file be $LOOPS_PATH. Definitions common to all steps are found in “shared_defs.xml”, described below.

```
<ROSETTASCRIPTS>

  {% include "shared_defs.xml" %}

  <MOVERS>
```

```
    <LoopModeler name="modeler"
      config="kic_with_frags"
      scorefxn_fa="scorefxn"
      loops_file="$LOOPS_PATH"
      fast="no">
        <Build skip="yes"/>
    </LoopModeler>
  </MOVERS>

  <PROTOCOLS>
    <Add mover_name="modeler"/>
    <Add mover_name="writer"/>
  </PROTOCOLS>

  <OUTPUT scorefxn="scorefxn"/>

</ROSETTASCRIPTS>
```

A representative command line for PIP Version 2 is shown below. Let the path to the "main" folder in Rosetta be */path/to/rosetta/main/*; the (relaxed) input PDB be $INPUT_PDB; the (unrelaxed) native PDB be $NATIVE_PDB; the folder where models are to be saved be $OUPUT_FOLDER; the name of the particular design be $OUTPUT_NAME; the "predict models" Rosetta script be *predict_models.xml*; the paths to the 9-mer fragments for the first and second loop be *path_to_9mers_A* and *path_to_9mers_B*, respectively, and the paths to 3-mer fragments for the first and second loop be *path_to_3mers_A* and *path_to_3mers_B*, respectively.

```
/path/to/rosetta/main/source/bin/rosetta_scripts.linuxgccrelease \
-database /path/to/rosetta/main/database/ \
-in:file:s $INPUT_PDB \
-in:file:native $NATIVE_PDB \
-out:prefix $OUTPUT_FOLDER \
-out:suffix $OUTPUT_NAME \
-out:no_nstruct_label -out:overwrite -out:pdb_gz \
-out:mute protocols.loops.loops_main \
-parser:protocol predict_models.xml \
-loops:frag_sizes 9 9 3 3 \
-loops:frag_files path_to_9mers_A path_to_9mers_B path_to_3mers_A path_to_3mers_B
```

PIP Version 2 Shared Definitions

Filter, scorefunction, residue selector, and certain Rosetta mover definitions were used during every step of the PIP Version 2 protocol. These were stored as separate RosettaScripts files and imported

into each main step template. Let the path to the scorefunction weights file be $SCOREFXN_WEIGHTS and the path to the constraints file be $CONSTRAINTS. The weights file was identical to the default weights for the ref2015 scorefunction.

```
{% include "filters.xml" %}

<SCOREFXNS>
  <ScoreFunction name="scorefxn" weights="$SCOREFXN_WEIGHTS"/>
  <ScoreFunction name="scorefxn_cst" weights="$SCOREFXN_WEIGHTS">
    <Reweight scoretype="coordinate_constraint" weight="1.0"/>
    <Reweight scoretype="atom_pair_constraint" weight="1.0"/>
    <Reweight scoretype="angle_constraint" weight="1.0"/>
    <Reweight scoretype="dihedral_constraint" weight="1.0"/>
    <Reweight scoretype="res_type_constraint" weight="1.0"/>
    <Reweight scoretype="chainbreak" weight="100.0"/>
  </ScoreFunction>
</SCOREFXNS>

<RESIDUE_SELECTORS>
  <Chain name="chA" chains="A"/>
  <Index name="E38" resnums="38"/>
</RESIDUE_SELECTORS>

<TASKOPERATIONS>
  <ReadResfile name="resfile"/>
  <ExtraRotamersGeneric name="ex" ex1="yes" ex2="yes" extrachi_cutoff="0"/>
  <LimitAromaChi2 name="aro" include_trp="yes"/>
  <IncludeCurrent name="curr"/>
</TASKOPERATIONS>

<MOVERS>
  <FavorNativeResidue name="nativebonus" />
  <ConstraintSetMover name="cst" cst_fa_file="$CONSTRAINTS"/>
  <WriteFiltersToPose name="writer" prefix="EXTRA_METRIC "/>
</MOVERS>
```

Filters for PIP Version 2

We used the following Rosetta script to run filters for all three steps in PIP Version 2, with the exceptions of the fragment quality metric (FragmentScoreFilter) and the Foldability metric, which were not included in the Structure Prediction step. For fragment picking, several variables are defined. Let the folder where fragment picking files are stored be $OUTPUT_DIR; the job-specific name of these files be $OUTPUT_NAME; the path to the required CSBLAST, BLAST, PSIPRED, and SPARKS-X

programs be */path/to/csblast-2.2.3_linux64*, */path/to/blast-2.2.26/bin/blastpgp*, */path/to/psipred/runpsipred_single*, and */path/to/sparks-x*, respectively; the path to the BLAST database consisting of FASTA-formatted sequence information for proteins in the PDB be */path/to/BLAST/sequences*; the weights file for scoring fragments be $FRAMGNET_WEIGHTS, and the path to the vall database */path/to/Rosetta/database/sampling/vall.jul19.2011.torsions.gz*.

```
<FILTERS>
  <PackStat
    name="PackStat Score [+]"
    threshold="0"
    chain="0"
    repeats="1"
  />
  <ResidueIE
    name="E38 Interaction Energy [-]"
    scorefxn="scorefxn_cst"
    score_type="total_score"
    energy_cutoff="-10"
    restype3="GLU"
    interface="0"
    whole_pose="0"
    selector="E38"
    jump_number="1"
    interface_distance_cutoff="8.0"
    max_penalty="1000.0"
    penalty_factor="1.0"
  />
  <PreProline
    name="Pre-Proline Potential [-]"
    use_statistical_potential="true"
  />
  <TotalSasa
    name="Total SASA [-]"
    threshold="0"
    upper_threshold="1000000000000000"
    hydrophobic="0"
    polar="0"
  />
  <ExposedHydrophobics
    name="Exposed Hydrophobic Residue SASA [-]"
    sasa_cutoff="20"
    threshold="-1"
  />
  <HbondsToResidue
```

```
    name="H-bonds to E38 [+]"
    scorefxn="scorefxn_cst"
    partners="0"
    energy_cutoff="-0.5"
    backbone="true"
    bb_bb="true"
    sidechain="true"
    residue="38"
    from_other_chains="true"
    from_same_chain="true"
  />
  <HbondsToResidue
    name="H-bonds to E38 (Backbone) [+]"
    scorefxn="scorefxn_cst"
    partners="0"
    energy_cutoff="-0.5"
    backbone="true"
    bb_bb="true"
    sidechain="false"
    residue="38"
    from_other_chains="true"
    from_same_chain="true"
  />
  <HbondsToResidue
    name="H-bonds to E38 (Sidechain) [+]"
    scorefxn="scorefxn_cst"
    partners="0"
    energy_cutoff="-0.5"
    backbone="false"
    bb_bb="false"
    sidechain="true"
    residue="38"
    from_other_chains="true"
    from_same_chain="true"
  />
  <BuriedUnsatHbonds
    name="Buried Unsatisfied H-Bonds [-]"
    scorefxn="scorefxn"
    print_out_info_to_pdb="true"
    task_operations="resfile"
  />
  <OversaturatedHbondAcceptorFilter
    name="Oversaturated H-bonds [-]"
    scorefxn="scorefxn_cst"
```

```
  max_allowed_oversaturated="0"
  hbond_energy_cutoff="-0.5"
  consider_mainchain_only="false"
/>
<RepackWithoutLigand
  name="Repack Without Ligand (delta REU) [-]"
  scorefxn="scorefxn_cst"
  target_res="all_repacked"
  rms_threshold="100"
/>
{% if w.focus_name != 'validate_designs' %}
<Foldability
  name="Foldability (35-41)"
  tries="60"
  start_res="35" {# Unaffected by loop length. #}
  end_res="41"   {# Unaffected by loop length. #}
/>
<Foldability
  name="Foldability (37-44)"
  tries="60"
  start_res="37" {# Unaffected by loop length. #}
  end_res="44"   {# Unaffected by loop length. #}
/>
<FragmentScoreFilter
  name="Max 9-Residue Fragment RMSD (C alpha) [-]"
  scoretype="FragmentCrmsd"
  sort_by="FragmentCrmsd"
  threshold="9999"
  direction="-"
  start_res="26 "
  end_res="51"
  compute="maximum"
  outputs_folder="$OUTPUT_DIR"
  outputs_name="$OUTPUT_NAME"
  csblast="/path/to/csblast-2.2.3_linux64"
  blast_pgp="/path/to/blast-2.2.26/bin/blastpgp"
  placeholder_seqs="/path/to/BLAST/sequences"
  psipred="/path/to/psipred/runpsipred_single"
  sparks-x="/path/to/sparks-x"
  sparks-x_query="/path/to/sparks-x/bin/buildinp_query.sh"
  frags_scoring_config="$FRAGMENTS_WEIGHTS"
  n_frags="200"
  n_candidates="1000"
  fragment_size="9"
```

```
    vall_path="/path/to/Rosetta/main/database/sampling/vall.jul19.2011.torsions.gz"
    print_to_pdb="true"
  />
  {% endif %}
</FILTERS>
```

The fragment quality filter required the following weights file describing which scores to use when picking fragments[8] during the structure prediction step:

```
# score name          priority  wght   min_allowed  extras
ProfileScoreL1           700     1.0     -
ProfileScoreStructL1     100     4.0     -
SolventAccessibility     500     1.5     -
Phi                      300     1.0     -
Psi                      200     0.6     -
SecondarySimilarity      600     1.0     -    predA
RamaScore                400     0.8     -    predA
FragmentCrmsd              0     0.0     -
```

EXPERIMENTAL CHARACTERIZATION OF DESIGNS

Purification of KSI designs

Cells were lysed in 40 mM potassium phosphate, 2 mM DTT, 1 mM EDTA, and 6 U/mL DNAse I, pH 7.2 using a Microfluidics M-110L microfluidizer. Clarified lysate was then passed through a 10 mL sodium deoxycholate gravity affinity column, prepared as described in reference[15]. The column was washed with 400 mM phosphate, 2 mM DTT, 1 mM EDTA, pH 7.2 followed by lysis buffer (minus DNAse), then eluted with 40 mM phosphate, 2 mM DTT, 1 mM EDTA, and 50% ethanol, pH 7.2. Proteins were then either further purified using a HiLoad 16/600 Superdex 75 pg gel filtration column or dialyzed twice in 1L lysis buffer to remove the ethanol. Most other designed proteins expressed in the insoluble fraction, so inclusion bodies were first purified from the cell lysate: Cells were grown in 1 L LB broth to an optical density of 0.6 at 37 °C, followed by overnight expression at 18 °C. Cells were then harvested by centrifugation at 3500 rpm for 20 minutes at 4 °C, then resuspended in lysis buffer (40 mM Tris-HCl, 1 mM EDTA, 25% sucrose w/v, pH 8.5). Suspensions were lysed in a M-110L microfluidizer and centrifuged at 20,000 rpm for 20 minutes at 4 °C. The resulting inclusion body pellet was washed once in 25 mL of 20 mM Tris-HCl, 1% sodium deoxycholate, 200 mM NaCl, and 2 mM EGTA, followed by at least 3 washes of 25 mL 10 mM Tris-HCl, 0.25% sodium doxycholate, pH 8.5, followed by at least 3 washes of 25 mL 20 mM Na-HEPES, 500 mM NaCl, 1 mM EDTA, pH 8.5. Inclusion bodies were centrifuged at 8,000 xg for 10 minutes at 4 °C between washes. Proteins in inclusion bodies were solubilized by shaking for 30 minutes with 10 mL 8 M urea, 20 mM Na-HEPES, 500 mM NaCl, 10 mM DTT, and 1 mM EDTA at pH 8.5, then centrifuged at 20,000 rpm for 20 minutes at 4 °C to remove cell debris. Solubilized protein was then refolded by stirring for 2 hours at 4 °C in 200 mL of 40 mM KPi, 1 mM EDTA, 2 mM DTT. Proteins were then sterile-filtered using 0.4 μm filter paper and further purified via deoxycholate column as described above.

Size exclusion chromatography

Six uM of purified wild-type KSI, V1D8r, or V2D9r were loaded onto a Superdex 75 10/300 GL column from Cytiva which was pre-equilibrated with running buffer (40 mM phosphate, 2 mM DTT, and 1 mM EDTA). Samples were run isocratically in an Agilent Technologies 1200 Series HPLC for 150 minutes and absorbance was monitored at 280 nm.

CD spectroscopy

Samples for CD analysis were prepared at approximately 6 μM enzyme in 40 mM phosphate pH 8.5, 2 mM DTT, and 1 mM EDTA. CD spectra were recorded at 25 °C using 2 mm cuvettes (Starna, 21-Q-2) in a JASCO J-710 CD spectrometer (Serial #9079119). The bandwidth was 2 nm, rate of scanning 20 nm/min, data pitch 0.2 nm, and response time 8 s. Each spectrum represents the average of 5 scans. Buffer spectra were subtracted from the sample spectra using the Spectra Manager software Version 1.53.01 from JASCO Corporation. Melting temperatures were assessed by measuring molar ellipticity at 222 nm and increasing the temperature from 25 °C to 95 °C at 1 °C per minute, using a data pitch of either 0.1, 0.5, or 1.0 °C.

X-ray data collection and processing

Prior to X-ray data collection, crystals were cryoprotected and flash-cooled by rapid plunging into liquid nitrogen. Crystals that yielded the V1D8r structure were cryoprotected using a mixture of 50% glycerol and 50% crystallization mother liquor, and crystals that yielded the V2D9r structure were cryoprotected using a mixture of 25% glycerol and 75% crystallization mother liquor. We collected single-crystal X-ray diffraction data on beamline 8.3.1 at the Advanced Light Source. Data collection for V1D8r was performed while the beamline was equipped with a Quantum 315r CCD detector (ADSC), while data collection for the V2D9r structure utilized a newer Pilatus3 S 6M photon-counting detector (Dectris). Both data sets were collected using an X-ray energy of 11111 keV, and the crystals were maintained at a cryogenic temperature (100 K) throughout the course of data collection.

We processed the X-ray data using the Xia2 system[16], which performed indexing, integration, and scaling with XDS and XSCALE[17], followed by merging with Pointless[18]. For the [6UAE] structure, a resolution cutoff (1.93 Å) was taken where the signal-to-noise ratio (<I/σI>) of the data fell to a value of approximately 1.0. In the case of the V1D8r structure, the data were collected on an older, smaller detector, and the resolution was limited by the detector edge and the geometric requirements of the experiment. Although other metrics of data quality (such as CC1/2 and <I/σI>) suggest that a more aggressive resolution cutoff would be acceptable, we were limited by the data completeness that could be obtained with the minimum accessible sample-to-detector distance. Further information regarding data collection and processing is presented in **Supplementary Table 13**. The reduced diffraction data were analyzed with phenix.xtriage to check for common crystal pathologies, none of which were identified.

SUPPLEMENTARY NOTE 1

Analysis of failure cases

Despite the performance improvements with FKIC, in particular for the challenging *Mixed Segment* and *Multiple Segments* datasets, FKIC failed to accurately model 12 of the 45 segments in the *Standard* 12-residue benchmark dataset. In four cases (1cs6, 1msc, 2tgi and 4i1b) no sub-Å model was generated. In the other eight cases (1arb, 1bhe, 1cyo, 1m3s, 1onc, 1qlw, 1t1d and 1thg), sub-Å models were generated but could not be identified by energy (the RMSDs of lowest energy structures were larger than 1.1Å). These failures could result from deficiencies in sampling near-native conformations, from inaccuracies in the energy function and/or from problems with the crystal structure conformation such as effects of crystal packing. Sampling and energy function errors are often coupled, as the energy function guides sampling during the simulations. To gain insights into potential reasons for the failures we observed, we ran simulations of the failed proteins starting from their native structures as inputs. In these simulations, we skipped the first build stage (yellow in **Supplementary Fig. 1**) so that the native bond angles and bond lengths were kept.

The results of these simulations allowed us to classify failure cases into 4 categories (**Supplementary Fig. 2**):

(1) Only the native-input simulations generate sub-Å models, which are correctly identified by energy. This occurred in two of the 12 failure cases (1cs6 and 2tgi). As the energies of native-like models are much lower than the non-native decoys (**Supplementary Fig. 2a**), failures in these cases are most likely due to the insufficient sampling.

(2) Both standard and native-input FKIC generate sub-Å models, but these models are only correctly identified by lowest energy in the native-input simulations (three of the 12 failure cases: 1bhe, 1onc and 1t1d). As the native-input simulations generated a larger number of correct models with lower energies (**Supplementary Fig. 2b**), the failures are likely caused by the failure of Rosetta to efficiently sample near-native energy minima. One of the possible explanations is that the standard simulation idealizes bond lengths and bond angles in standard FKIC. Because of the rugged energy landscape, small conformational changes can result in significant energy differences.

(3) As in (2), both simulations generate sub-Å models and native-input simulations correctly identify these models by energy, but standard FKIC generates incorrect models with lower energies (**Supplementary Fig. 2c,** two of the 12 failure cases: 1arb and 1qlw). This behavior indicates linked scoring and sampling deficiencies.

(4) Neither standard nor native input simulations generate sub-Å models (**Supplementary Fig. 2d**; five of 12 failure cases: 1cyo, 1m3s, 1msc, 1thg and 4i1b). While these simulations start from the native backbones, they do not include crystal contacts. Because crystal packing affecting loop conformations

is well known[19,20], there is a formal possibility that the failures of 1cyo, 1m3s, 1msc and 4i1b are due to crystal packing. For the lowest energy models for 1cyo and 4i1b, the incorrectly modeled loops would have unfavorable contacts in the crystal lattice. For 1m3s and 1msc, the native loop conformations make contacts with another monomer in the crystal. For 1thg, the RMSD of the lowest energy structure improved from 1.86Å to 1.12Å when including native bond lengths and angles, so there might be both sampling and energy function problems.

In sum, in particular for categories (1) and (2), it could be beneficial to incorporate sampling of bond lengths and bond angles, which we kept to their idealized values to reduce the conformational space to be sampled. Category (3) is indicative of energy function failures although we note that sampling and scoring are coupled in our simulations that accept or reject models based on their energies. Category (4) identifies a number of cases where crystal packing may influence the conformation of the modeled segment in the experimentally determined structure.

SUPPLEMENTARY NOTE 2

8-Residue and 12-residue *Multiple Segments* datasets

To further benchmark the performance of FKIC on multiple interacting segments, we constructed a dataset of 8-residue interacting segments and a dataset of 12-residue interacting segments in a similar manner to the construction of the 10-residue *Multiple Segments* dataset (see **Supplementary Methods**). On the 8-residue interacting segment dataset, FKIC has 0.65Å median accuracy and 59.9% median fraction of sub-Å prediction; NGK has 0.79Å median accuracy and 35.6% median fraction of sub-Å prediction (median accuracy and median fraction of sub-Å prediction are described in the main text). On the 12-residue interacting dataset, FKIC has 1.53Å median accuracy and 0.21% median fraction of sub-Å prediction; NGK has 1.94Å median accuracy and 0% median fraction of sub-Å prediction. Thus, FKIC improves the prediction accuracy for multiple interacting segments consistently on different segment lengths and is able to find correct solutions for large conformational search problems, such as the set with two interacting 12-residue segments where previous methods such as NGK and CCD frequently fail (**Supplementary Table 6** and **Supplementary Table 7**).

SUPPLEMENTARY NOTE 3

Benchmark LHKIC on structure prediction

The LHKIC method was developed for loop design, but it can also be used for structure prediction. We benchmarked the prediction performance of LHKIC on all three benchmark datasets (**Supplementary Table 1**). The performance of LHKIC is comparable to NGK and FKIC in terms of median RMSD of lowest scoring models. For the *Standard* and *Mixed Segment* datasets, the median sub-Å fraction of LHKIC is 24.35% and 15.40%, better than NGK but worse than FKIC. This result indicates that sequence-independent fragments (as in LHKIC) can improve sampling in structure predictions over non fragment-based methods such as NGK, but the improvement is smaller than when using fragments picked with sequence information as in FKIC (and for the *Multiple Segments* dataset, LHKIC had a median sub-Å fraction of 4.01%, which is lower than for both NGK and FKIC.) Note that the absolute energies for LHKIC cannot be directly compared to the other methods since LHKIC was developed in a newer version of Rosetta (revision 60022, see **Methods**).

SUPPLEMENTARY NOTE 4

Catalytic activity of KSI designs

V1D8r and V2D9r were overall less active than both wild-type and D38E KSI (**Table 1**). This result might be expected for several reasons: First, while we took steps to avoid mutations in residues known to be important for catalysis, we still made extensive changes (19 and 12 mutations in design V1D8r and V2D9r, respectively) in and around the active site, which could change the electrostatic environment as well as affect functional or non-productive dynamics that impact catalysis. Second, while wild-type KSI is a dimer, our designs (while modeled as a dimer) are monomeric at the concentrations of the enzyme assay (**Supplementary Fig. 5**) although dimeric in the crystal. These differences could affect functional group positioning in solution[21]. Third, even though the glutamate side chain placement in V2D9r was close to ideal, it was not perfect; even small perturbations towards nonproductive conformations can be significant to catalysis and the designed glutamate may only sample catalytic conformations a fraction of the time. Finally, there is evidence that the catalytic residue in the homologous *Pseudomonas putida* KSI accesses multiple specific productive conformations to enable its participation throughout the catalytic cycle[22], a property which was not considered by our protocol. Despite these difficulties, our designed enzymes still enhanced the catalysis of their substrate by 4 to 5 orders of magnitude when compared to the water-catalyzed isomerization of the similar 5-androstene-3,17-dione[23].

SUPPLEMENTARY NOTE 5

Analysis of sequence differences between wildtype KSI and V2D9r

The mutational effects of alanine and wildtype reversion mutations on catalytic activity of V2D9r are all rather small, except for mutations at the catalytic E38 to either aspartate or alanine. Most of the other changes are within error (**Fig. 4f**, shaded bar). Exceptions are Q41A that has slightly decreased activity and T39S that has a slightly increased activity. Q41 is the residue with the largest shift in Cα position between aligned wildtype and V2D9r crystal structures (**Supplementary Table 14**). T39S is a manually designed mutation to avoid a potential issue with burial of polar groups. Nevertheless, none of the individual side chain mutations (apart from mutations to the catalytic glutamate) show more than 2-fold effects on kinetics. This observation suggests that there are not individual key side chain-mediated interactions that determine the new loop conformation, but that many interactions each contribute to a smaller extent.

We also analyzed the local sequence-structure compatibility of the sequences of wildtype and V2D9r with the two structures in the reshaped segment using the Rosetta fragment quality metric (**Supplementary Methods**). This metric does not consider specific side chain interactions but instead evaluates a tertiary-structure-independent compatibility of the primary sequence for a given structure. **Supplementary Figure 6f** shows that the wild-type sequence has a lower (better) fragment RMSD for the wild-type structure at all positions in the reshaped region, whereas the sequence of V2D9r has a lower fragment RMSD for the designed structure at most positions in the designed regions. These observations are consistent with the idea that the structure of V2D9r in the reshaped region is at least partially dependent on local sequence-structure compatibility in addition to tertiary interactions.

SUPPLEMENTARY NOTE 6

Loop modeling on template-based models and perturbed datasets

To test the performance of FKIC in contexts where the environment of the remodeled loop is non-native, we benchmarked FKIC on several datasets from ref[24]: a benchmark set from template-based modeling, and three sidechain/backbone perturbed loop datasets (**Supplementary Table 11**). On the side chain perturbed datasets, FKIC performs similarly to NGK, and outperforms the other methods. This behavior is likely due to the fact that surrounding residues are repacked during FKIC or NGK simulations, indicating that these methods can account for slight imperfections in the environment that can be resolved by altering side chain conformations. On the backbone-perturbed dataset, the performance of FKIC is comparable to the reported results of GalaxyLoop-PS2[24], with a median RMSD of lowest-scoring models of 1.68Å and 1.65Å for FKIC and Galaxy-PS2, respectively. On the template-based model dataset, none of the methods performs well, with the median RMSD of lowest scoring models above 3 Å for all methods.

It should be noted that the prediction and evaluation approach described here is not suited to appropriately assess the accuracy of loop modeling methods in environments where the surrounding backbone is perturbed, since the backbone in the environment is not allowed to change during the simulation. In our study, we compare a predicted loop structure to the native loop structure after aligning the surrounding environment of the loop. The native loop is the correct answer when the surrounding environment is unperturbed. However, the native loop conformation may not be compatible with the perturbed backbone and can therefore not be identified as the lowest scoring model in simulations where the backbone of the environment remains in its (unchanged) perturbed conformation. Our analysis on the dataset containing backbone perturbations after MD simulations from ref[24] supports this argument. For each protein in this dataset, we defined the residues within 10 Å from the native loop as surrounding residues. We then superimposed the native structure and the perturbed structure by the backbone heavy atoms of the surrounding residues and calculated heavy atom steric clashes between the native loop and the perturbed surrounding backbone atoms. Two heavy atoms were defined as clashing if their interatomic distance was within 2.5 Å. This analysis revealed that twelve out of the twenty native loops in the dataset contained clashes with their perturbed environments that cannot be resolved with simulations that do not relax the surrounding backbone. When excluding these cases, the FKIC and GalaxyLoop-PS2 median RMSDs improved to 1.3 Å and 1.4 Å, respectively (**Supplementary Table 11**).

These considerations highlight that modeling loops in perturbed environments such as homology models remains an important unsolved problem. To adapt the design-centric loop modeling methods presented here to the problem of homology modeling should include simultaneous or iterative refinement of both loop structures and the environment.

SUPPLEMENTARY TABLES

Supplementary Table 1. Datasets and performance summary

a) Comparison of methods

Dataset	Sampling method	Rosetta energy function	Median RMSD of lowest energy model (Å)	Median RMSD of lowest RMSD model (Å)	Median RMSD all models (Å)	Median sub-A fraction	Median lowest energy (REU)	Median time (s)
Standard	KIC*	score12	1.05	NA	NA	4.30%	NA	NA
Standard	CCD	ref2015	1.26	0.47	3.27	2.00%	-709.18	2299
Standard	NGK	ref2015	0.64	0.37	2.70	13.00%	-712.65	3642
Standard	FKIC	ref2015	0.62	**0.32**	**1.16**	**47.80%**	**-716.78**	3456
Standard	LHKIC	ref2015	**0.55****	0.34	2.66	24.35%	-655.18***	3057
Mixed	CCD	ref2015	1.29	0.67	3.46	0.50%	-719.42	4309
Mixed	NGK	ref2015	1.07	0.45	4.65	1.15%	-728.18	7341
Mixed	FKIC	ref2015	0.53	**0.34**	**1.46**	**52.30%**	**-739.38**	7196
Mixed	LHKIC	ref2015	**0.48**	0.35	4.14	15.40%	-693.95***	5322
Multiple	CCD	ref2015	1.97	0.90	2.95	0.20%	-557.06	5204
Multiple	NGK	ref2015	1.29	0.52	2.35	5.50%	-573.60	9472
Multiple	FKIC	ref2015	**1.00**	**0.41**	**1.82**	**28.50%**	**-581.42**	8834
Multiple	LHKIC****	ref2015	1.31	0.55	2.50	4.01%	-517.69***	7831

REU, Rosetta energy units
* taken from ref.[1]; all other simulations were run using the Rosetta energy function “ref2015” as described in ref.[25]
** bold numbers denote best performance for given dataset
*** REU value not directly comparable to other methods as LHKIC was benchmarked using a more recent Rosetta version (see **Methods**).
**** PDB 1FO9 was excluded from the *Multiple Segments* benchmark set for LHKIC because for this case simulations failed to converge during minimization for the majority of simulations.

b) Inclusion of fragments from homologous structures

Dataset	Sampling method	Rosetta energy function	Median RMSD of lowest scoring model (Å)	Median RMSD of lowest RMSD model (Å)	Median RMSD all models (Å)	Median sub-A fraction	Median lowest energy (REU)	Median time (s)
Standard	FKIC	talaris2013	0.70	0.36	1.19	44.89%	-274.50	1646
Standard	FKIC (+ homologs)	talaris2013	**0.59**	**0.33**	**0.85**	**66.80%**	-273.29	1919

Supplementary Table 2. *Mixed Segment* dataset detailed performance

		CCD					NGK					FKIC				
**PDB name**	Target segment residues	RMSD of lowest energy model (Å)	Lowest energy (REU)	Lowest RMSD (Å)	Energy of lowest RMSD model (REU)	Fraction sub-Å models	RMSD of lowest energy model (Å)	Lowest energy (REU)	Lowest RMSD (Å)	Energy of lowest RMSD model (REU)	Fraction sub-Å models	RMSD of lowest energy model (Å)	Lowest energy (REU)	Lowest RMSD (Å)	Energy of lowest RMSD model (REU)	Fraction sub-Å models
**1a8d**	275-290	3.04	-984.24	1.94	-944.26	0.00	2.94	-992.55	0.77	-984.31	0.01	0.42	-1000.57	0.38	-974.06	0.04
**1arb**	165-180	4.80	-510.37	3.21	-493.67	0.00	6.23	-528.00	1.17	-507.84	0.00	6.48	-531.35	2.31	-509.84	0.00
**1bhe**	340-355	2.10	-820.58	0.72	-815.20	0.00	0.66	-843.95	0.53	-824.24	0.36	0.76	-835.54	0.65	-825.45	0.07
**1bn8**	38-53	3.08	-937.27	1.37	-928.40	0.00	0.55	-946.28	0.44	-941.02	0.03	0.52	-948.32	0.45	-943.16	0.03
**1c5e**	57-72	2.77	-691.08	1.33	-524.80	0.00	3.57	-706.51	0.50	-699.10	0.03	0.29	-709.42	0.29	-709.42	0.67
**1cb0**	91-106	0.63	-662.32	0.47	-651.31	0.28	0.68	-668.11	0.30	-663.53	0.97	0.61	-683.38	0.34	-663.29	0.96
**1cs6**	46-61	5.27	-779.57	0.87	-766.75	0.00	1.34	-789.74	0.74	-781.50	0.00	1.27	-796.08	0.51	-770.26	0.16
**1dqz**	103-118	0.50	-1243.04	0.36	-1207.68	0.33	2.40	-1255.72	0.45	-1222.43	0.01	0.35	-1261.68	0.24	-1198.37	0.84
**1ede**	76-91	0.38	-694.76	0.38	-694.76	0.03	0.26	-710.85	0.19	-689.15	0.37	0.28	-719.71	0.21	-706.81	0.72
**1exm**	252-267	1.45	-977.38	0.66	-969.81	0.01	4.62	-983.36	1.38	-962.42	0.00	4.38	-991.09	0.38	-983.50	0.21
**1ezm**	24-39	6.88	-654.25	3.31	-556.10	0.00	0.50	-684.52	0.40	-668.73	0.00	6.00	-681.88	1.37	-611.59	0.00
**1f46**	45-60	5.48	-705.82	1.07	-701.81	0.00	7.10	-723.09	0.99	-708.18	0.00	1.51	-735.38	1.08	-704.28	0.00
**1i7p**	155-170	0.68	-647.33	0.68	-647.33	0.00	1.59	-654.72	1.51	-648.48	0.00	0.56	-657.64	0.37	-652.65	0.46
**1ms9**	431-446	10.98	-2721.15	4.21	-2392.51	0.00	11.83	-2744.95	3.10	-2427.70	0.00	6.42	-2749.58	0.70	-2079.87	0.01
**1oth**	272-287	1.31	-612.54	0.74	-510.97	0.01	2.24	-685.23	0.45	-555.42	0.02	0.71	-673.30	0.38	-559.34	0.80
**1oyc**	330-345	1.35	-557.96	0.48	-137.82	0.08	5.66	-536.60	0.59	126.42	0.00	0.54	-593.99	0.39	-471.11	0.65
**1pbe**	200-215	0.35	-803.31	0.35	-803.31	0.17	0.59	-814.05	0.31	-807.25	0.25	0.57	-816.86	0.25	-813.62	0.58
**1qlw**	284-299	0.46	-1557.59	0.39	-1547.37	0.09	0.52	-1566.76	0.34	-1561.36	0.06	0.35	-1573.51	0.18	-1569.30	0.73
**1srp**	418-433	0.53	-733.70	0.53	-733.70	0.00	0.67	-733.27	0.65	-729.35	0.01	0.42	-743.39	0.29	-739.06	0.32
**1tca**	163-178	2.33	-829.93	1.05	-815.78	0.00	0.28	-850.81	0.28	-850.81	0.08	0.36	-852.46	0.27	-850.07	0.17
**1thg**	359-374	3.78	-1290.91	0.98	-798.31	0.00	7.31	-836.82	2.20	-782.91	0.00	0.87	-820.01	0.76	-793.95	0.28
**1thw**	97-112	0.50	-365.34	0.50	-365.34	0.00	0.33	-404.36	0.28	-361.20	0.35	0.34	-406.53	0.27	-361.89	0.61
**1tib**	166-181	0.49	-469.26	0.49	-469.26	0.07	0.30	-513.50	0.25	-488.46	0.71	0.29	-512.87	0.25	-499.93	0.81
**1tml**	26-41	0.83	-733.03	0.69	-729.62	0.02	0.35	-756.99	0.34	-745.83	0.04	0.35	-764.53	0.26	-756.61	0.80
**1xif**	50-65	1.28	-815.74	0.47	-813.47	0.02	0.45	-820.65	0.37	-819.84	0.01	0.59	-829.72	0.32	-820.15	0.35
**2ebn**	11-26	0.50	-686.47	0.50	-686.47	0.03	0.31	-715.52	0.25	-708.04	0.09	0.29	-719.70	0.24	-711.08	0.74
**2exo**	48-63	3.51	-615.43	1.17	-602.03	0.00	4.79	-654.47	2.30	-602.47	0.00	4.88	-680.12	0.43	-626.40	0.07
**2pia**	43-58	1.08	-666.90	0.37	-594.66	0.33	1.52	-683.56	0.39	-581.39	0.43	0.45	-682.60	0.34	-600.69	0.96
**2sil**	209-224	0.65	-440.99	0.35	-366.67	0.13	6.82	-523.38	0.37	-370.74	0.01	0.48	-551.19	0.29	-365.12	0.67
**3hsc**	125-140	0.33	-874.75	0.31	-869.11	0.99	0.81	-868.57	0.81	-868.57	0.01	0.30	-878.17	0.27	-873.40	0.80

Supplementary Table 3. *Multiple Segments* dataset detailed performance

		CCD					NGK					FKIC				
**PDB name**	Target segment residues	RMSD of lowest energy model (Å)	Lowest energy (REU)	Lowest RMSD (Å)	Energy of lowest RMSD model (REU)	Fraction sub-Å models	RMSD of lowest energy model (Å)	Lowest energy (REU)	Lowest RMSD (Å)	Energy of lowest RMSD model (REU)	Fraction sub-Å models	RMSD of lowest energy model (Å)	Lowest energy (REU)	Lowest RMSD (Å)	Energy of lowest RMSD model (REU)	Fraction sub-Å models
**1a3a**	11-20<br>87-96	5.65	-340.13	0.83	-284.99	0.00	0.84	-316.80	0.45	-246.79	0.13	0.84	-325.15	0.48	-254.18	0.58
**1deu**	170-179<br>225-234	6.49	16511.10	1.81	16591.50	0.00	2.21	16114.00	1.56	16135.00	0.00	2.57	16115.50	0.97	16143.30	0.00
**1dqz**	148-157<br>252-261	2.62	-623.02	1.45	-555.00	0.00	1.93	-671.27	0.41	-637.28	0.12	2.07	-667.08	0.55	-626.29	0.06
**1euv**	529-538<br>570-579	2.17	-500.01	1.56	-474.99	0.00	1.88	-521.07	0.96	-513.85	0.00	1.10	-523.17	0.93	-517.57	0.01
**1fo9**	239-248<br>261-270	1.09	-774.02	1.09	-774.02	0.00	0.84	-804.34	0.43	-795.79	0.67	0.81	-811.44	0.40	-797.06	0.83
**1ftr**	211-220<br>278-287	9.14	-700.66	2.23	-670.62	0.00	8.51	-719.42	2.41	-696.58	0.00	3.02	-714.95	0.77	-712.83	0.00
**1h1n**	116-125<br>153-162	0.49	-651.04	0.43	-637.42	0.17	1.29	-670.91	0.25	-633.85	0.36	0.36	-671.26	0.23	-668.07	0.60
**1h6u**	170-179<br>202-211	0.37	-728.01	0.36	-724.78	0.45	0.37	-732.42	0.32	-729.53	0.28	0.42	-734.81	0.28	-730.74	0.38
**1i7k**	85-94<br>118-127	3.33	-308.26	1.79	-295.82	0.00	3.11	-326.13	1.51	-317.13	0.00	1.95	-325.51	1.06	-308.24	0.00
**1idp**	71-80<br>111-120	0.65	-289.74	0.44	-286.54	0.11	4.43	-302.14	0.60	-296.58	0.02	0.77	-306.48	0.28	-296.60	0.80
**1inl**	251-260<br>266-275	2.65	-520.07	0.67	-432.87	0.00	0.52	-579.68	0.43	-562.67	0.07	0.57	-586.58	0.40	-558.11	0.55
**1j7d**	35-44<br>69-78	0.61	-332.51	0.47	-324.83	0.04	1.30	-337.14	1.01	-328.33	0.00	2.01	-341.38	0.93	-329.21	0.02
**1jfr**	44-53<br>15-24	3.61	-554.55	0.71	-434.64	0.01	1.28	-567.51	0.38	-469.28	0.42	7.05	-576.27	0.36	-494.83	0.17
**1jfu**	34-43<br>136-145	1.38	-407.70	0.91	-398.11	0.00	5.58	-414.98	1.04	-403.42	0.00	1.19	-415.17	0.91	-397.13	0.02
**1ku1**	628-637<br>645-654	0.67	550.86	0.58	551.87	0.22	0.35	445.64	0.35	542.16	0.22	0.40	445.59	0.27	455.27	0.83
**1kzq**	44-53<br>83-92	3.58	-444.31	1.87	-407.29	0.00	2.61	-466.18	1.72	-458.54	0.00	1.99	-463.25	0.85	-450.18	0.00
**1m0z**	71-80<br>49-58	0.72	-559.57	0.57	-550.88	0.15	0.73	-564.57	0.51	-558.06	0.05	0.66	-571.10	0.40	-560.56	0.76
**1nxm**	12-21<br>98-107	0.40	-465.63	0.24	-453.77	0.47	0.32	-482.36	0.23	-468.24	0.92	0.24	-476.32	0.22	-468.21	0.98
**1qwd**	18-27<br>93-102	2.97	-376.26	1.84	-368.08	0.00	1.17	-393.42	0.78	-388.26	0.00	0.90	-389.35	0.36	-383.74	0.01
**1t6g**	140-149<br>197-206	1.00	-753.66	0.81	-737.17	0.01	1.17	-777.29	0.58	-753.35	0.17	1.10	-777.69	0.56	-763.90	0.44
**1u09**	240-249<br>293-302	1.85	-916.14	1.23	-893.76	0.00	2.13	-1110.23	1.00	-902.43	0.00	3.31	-933.51	0.39	-926.61	0.01
**1w0d**	275-284<br>315-324	1.84	-663.35	0.86	-662.27	0.00	1.61	-670.13	0.54	-664.68	0.09	0.75	-669.27	0.41	-662.14	0.66

		CCD					NGK					FKIC				
**PDB name**	Target segment residues	RMSD of lowest energy model (Å)	Lowest energy (REU)	Lowest RMSD (Å)	Energy of lowest RMSD model (REU)	Fraction sub-Å models	RMSD of lowest energy model (Å)	Lowest energy (REU)	Lowest RMSD (Å)	Energy of lowest RMSD model (REU)	Fraction sub-Å models	RMSD of lowest energy model (Å)	Lowest energy (REU)	Lowest RMSD (Å)	Energy of lowest RMSD model (REU)	Fraction sub-Å models
**1xdw**	49-58<br>27-36	0.89	-783.62	0.89	-783.62	0.00	0.68	-805.64	0.59	-797.40	0.42	0.68	-811.82	0.42	-802.75	0.62
**1xg2**	184-193<br>218-227	3.59	-594.97	0.93	-554.27	0.00	2.33	-637.06	0.49	-614.76	0.01	0.52	-638.36	0.41	-621.65	0.56
**1xsz**	77-86<br>119-128	0.91	-815.59	0.41	-774.90	0.57	0.49	-816.42	0.32	-793.45	0.35	0.58	-823.76	0.32	-795.46	0.95
**1xwt**	333-342<br>369-378	2.10	-941.99	0.92	-923.93	0.00	1.57	-976.37	0.35	-953.47	0.44	1.82	-977.69	0.40	-950.40	0.34
**1yif**	176-185<br>144-153	3.06	-748.46	2.18	-682.37	0.00	2.50	-761.17	1.81	-759.79	0.00	2.92	-765.53	1.88	-755.31	0.00
**1zvt**	659-668<br>716-725	3.72	-508.86	2.52	-505.57	0.00	0.32	-542.65	0.32	-542.65	0.07	1.49	-535.43	1.28	-531.60	0.00
**2a4a**	37-46<br>18-27	2.44	-598.61	1.60	-450.13	0.00	2.01	-620.40	0.71	-594.80	0.00	1.84	-631.78	0.93	-610.00	0.00
**2b0a**	102-111<br>123-132	0.45	-395.53	0.45	-395.53	0.00	0.42	-404.93	0.42	-404.93	0.04	0.38	-408.45	0.31	-395.35	0.24

Supplementary Table 4. *Standard* dataset detailed performance

		CCD					NGK					FKIC				
**PDB name**	Target segment residues	RMSD of lowest energy model (Å)	Lowest energy (REU)	Lowest RMSD (Å)	Energy of lowest RMSD model (REU)	Fraction sub-Å models	RMSD of lowest energy model (Å)	Lowest energy (REU)	Lowest RMSD (Å)	Energy of lowest RMSD model (REU)	Fraction sub-Å models	RMSD of lowest energy model (Å)	Lowest energy (REU)	Lowest RMSD (Å)	Energy of lowest RMSD model (REU)	Fraction sub-Å models
**1a8d**	155-166	2.83	-1008.38	2.14	-973.52	0.00	0.38	-1024.27	0.38	-1024.27	0.03	0.42	-1022.05	0.31	-1019.72	0.01
**1arb**	182-193	2.37	-554.35	0.23	-530.49	0.01	0.54	-562.94	0.37	-539.98	0.38	2.06	-563.70	0.39	-526.90	0.17
**1bhe**	121-132	2.09	-923.69	1.84	-913.87	0.00	0.68	-908.66	0.30	-903.45	0.18	1.91	-915.76	0.31	-906.99	0.13
**1bn8**	298-309	0.70	-987.69	0.33	-982.93	0.02	0.75	-993.21	0.43	-986.78	0.09	0.48	-1000.64	0.30	-991.29	0.44
**1c5e**	82-93	0.40	-742.69	0.30	-742.50	0.09	0.36	-745.93	0.28	-742.69	0.41	0.38	-748.17	0.31	-744.34	0.90
**1cb0**	33-44	0.41	-679.12	0.30	-671.31	0.10	0.25	-679.95	0.16	-651.78	0.36	0.36	-680.65	0.14	-675.44	0.84
**1cnv**	188-199	3.09	-691.00	1.24	-665.03	0.00	1.06	-701.43	0.47	-685.34	0.01	1.02	-700.87	0.39	-699.85	0.03
**1cs6**	145-156	3.60	-731.51	1.71	-704.86	0.00	4.26	-777.82	0.99	-760.81	0.00	4.24	-773.94	1.55	-745.58	0.00
**1cyo**	12-23	0.91	-192.93	0.58	-191.35	0.03	4.98	-197.51	0.53	-189.01	0.02	5.09	-196.90	0.51	-191.38	0.01
**1dqz**	209-220	0.40	-1471.41	0.40	-1471.41	0.00	0.49	-1476.24	0.30	-1456.86	0.33	0.46	-1480.22	0.32	-1325.32	0.32
**1dts**	41-52	7.66	-445.87	0.95	-436.78	0.00	6.63	-470.55	0.94	-436.96	0.01	1.07	-472.01	0.75	-417.87	0.16
**1eco**	35-46	0.36	-283.70	0.29	-267.09	0.63	0.40	-284.48	0.32	-277.13	0.49	0.37	-285.75	0.30	-269.98	0.99
**1ede**	150-161	1.26	-598.32	0.76	-592.01	0.01	0.72	-615.96	0.59	-612.93	0.05	0.77	-631.71	0.54	-613.56	0.21
**1exm**	291-302	0.52	-1062.06	0.47	-1051.67	0.35	0.47	-1048.50	0.27	-1040.46	0.62	0.63	-1048.74	0.27	-1039.80	0.89
**1ezm**	122-133	2.53	-733.12	1.25	-719.78	0.00	0.37	-758.95	0.37	-758.95	0.04	0.62	-753.62	0.44	-747.20	0.03
**1f46**	64-75	2.27	-709.18	0.80	-697.38	0.03	3.09	-712.65	0.78	-705.47	0.14	0.44	-716.78	0.32	-710.87	0.30
**1i7p**	63-74	0.51	-669.97	0.36	-661.74	0.11	0.42	-674.13	0.31	-651.77	0.53	0.38	-672.19	0.33	-669.48	0.90
**1m3s**	68-79	5.40	-672.68	0.68	-666.48	0.01	5.05	-674.66	0.44	-664.02	0.31	5.66	-673.18	0.42	-664.04	0.51
**1ms9**	529-540	2.48	-3018.90	0.77	-2998.79	0.04	0.27	-3041.97	0.22	-3041.75	0.91	0.35	-3044.20	0.23	-3030.97	0.69
**1msc**	9-20	7.68	151.93	2.93	188.30	0.00	6.54	148.08	1.27	162.88	0.00	7.85	147.53	1.08	155.56	0.00
**1my7**	254-265	0.49	-504.92	0.35	-496.96	0.11	0.61	-511.55	0.40	-507.90	0.75	0.53	-512.36	0.45	-507.57	0.81
**1onc**	23-34	2.88	-200.66	1.70	-190.59	0.00	3.63	-209.76	0.46	-208.03	0.04	3.62	-208.46	0.93	-200.42	0.00
**1oth**	69-80	0.52	-837.57	0.38	-836.26	0.18	0.45	-837.27	0.31	-834.46	0.34	0.50	-843.18	0.27	-833.69	0.97
**1oyc**	203-214	2.99	-776.66	0.89	-691.71	0.00	0.30	-818.69	0.28	-719.38	0.13	0.32	-819.70	0.25	-727.24	0.54
**1pbe**	129-140	2.60	-833.07	0.43	-790.79	0.06	0.45	-820.65	0.32	-801.51	0.32	0.38	-824.99	0.33	-804.46	0.72
**1qlw**	31-42	4.34	-1611.33	2.07	-1604.34	0.00	4.87	-1624.91	0.30	-1605.29	0.13	2.83	-1626.69	0.33	-1620.54	0.06
**1rro**	17-28	0.44	-272.26	0.34	-270.03	0.08	0.64	-277.16	0.39	-270.25	0.10	0.63	-279.08	0.32	-269.39	0.48
**1srp**	311-322	0.48	-782.41	0.31	-773.57	0.05	0.31	-877.44	0.25	-772.31	0.99	0.37	-879.03	0.25	-759.90	0.98
**1t1d**	127-138	0.42	-206.93	0.34	-204.69	0.14	2.79	-217.61	0.32	-207.60	0.09	2.94	-215.11	0.30	-209.60	0.39
**1tca**	305-316	9.52	-846.00	2.37	-806.46	0.00	0.66	-859.34	0.26	-854.54	0.12	0.29	-864.98	0.24	-848.65	0.24
**1thg**	127-138	2.88	-1419.28	1.64	-1385.22	0.00	1.05	-1434.24	0.71	-1411.26	0.07	1.86	-1434.72	0.28	-1423.84	0.07
**1thw**	178-189	1.06	-394.74	1.06	-394.74	0.00	0.65	-413.28	0.49	-411.02	0.05	0.61	-414.92	0.46	-409.31	0.23
**1tib**	99-110	0.64	-527.93	0.39	-524.55	0.10	0.75	-529.58	0.40	-526.29	0.07	0.63	-535.99	0.39	-529.46	0.57
**1tml**	243-254	0.46	-766.55	0.38	-765.86	0.05	0.50	-776.42	0.25	-773.23	0.51	0.52	-777.92	0.28	-774.05	0.79
**1xif**	203-214	1.90	-821.11	1.26	-814.10	0.00	0.41	-838.28	0.18	-828.66	0.94	0.37	-839.04	0.22	-833.59	0.85
**2cpl**	145-156	0.60	-457.29	0.26	-453.37	0.26	0.32	-459.00	0.21	-451.22	0.26	0.30	-458.11	0.24	-454.07	0.99
**2ebn**	136-147	0.35	-722.21	0.35	-722.21	0.02	0.44	-719.92	0.43	-719.89	0.01	0.60	-721.73	0.43	-718.80	0.09
**2exo**	293-304	0.61	-737.11	0.41	-728.13	0.12	0.49	-731.90	0.42	-722.45	0.18	0.47	-734.53	0.35	-725.14	0.99
**2pia**	30-41	1.00	-686.94	0.38	-662.42	0.55	0.82	-688.54	0.48	-680.08	0.99	0.86	-688.99	0.56	-673.23	1.00
**2rn2**	90-101	1.41	-283.79	1.41	-283.79	0.00	0.64	-297.34	0.32	-289.54	0.40	0.68	-298.25	0.29	-290.57	0.69
**2sil**	255-266	0.69	-744.26	0.41	-732.81	0.55	1.26	-745.15	0.60	-734.86	0.00	0.93	-747.04	0.46	-740.32	0.57
**2tgi**	48-59	2.69	-140.59	1.73	-121.99	0.00	3.57	-162.15	0.36	-89.29	0.00	3.14	-163.39	1.36	-87.48	0.00
**3cla**	176-187	1.32	-475.15	0.41	-472.78	0.21	1.21	-483.29	0.39	-478.16	0.04	0.64	-484.91	0.28	-477.79	0.63
**3hsc**	72-83	0.51	-894.79	0.46	-891.28	0.04	0.32	-896.76	0.25	-890.33	0.72	0.56	-899.54	0.25	-893.37	0.94
**4i1b**	46-57	3.63	-134.68	2.14	-130.51	0.00	6.69	-140.23	0.82	-135.44	0.00	6.63	-140.98	1.04	-97.29	0.00

Supplementary Table 5. Failure cases, *Standard* dataset

		FKIC				FKIC native input				Reason for failure
**PDB name**	Target segment residues	RMSD of lowest energy model (Å)	Lowest energy (REU)	Lowest RMSD (Å)	Energy of lowest RMSD model (REU)	RMSD of lowest energy model (Å)	Lowest energy (REU)	Lowest RMSD (Å)	Energy of lowest RMSD model (REU)	
**1arb**	182-193	2.06	-563.70	0.39	-526.90	0.40	-557.64	0.21	-537.67	insufficient sampling
**1bhe**	121-132	1.91	-915.76	0.31	-906.99	0.24	-940.55	0.24	-928.05	insufficient sampling
**1cs6**	145-156	4.24	-773.94	1.55	-745.58	0.39	-809.13	0.25	-804.74	insufficient sampling
**1cyo**	12-23	5.09	-196.90	0.51	-191.38	**1.06***	-194.88	0.32	-188.95	crystal packing
**1m3s**	68-79	5.66	-673.18	0.42	-664.04	**5.39**	-680.83	0.43	-669.76	crystal packing
**1msc**	9-20	7.85	147.53	1.08	155.56	**8.74**	146.72	0.86	163.39	crystal packing
**1onc**	23-34	3.62	-208.46	0.93	-200.42	0.51	-208.74	0.39	-200.49	insufficient sampling
**1qlw**	31-42	2.83	-1626.69	0.33	-1620.54	0.48	-1623.97	0.29	-1595.85	insufficient sampling / energy function deficiency
**1t1d**	127-138	2.94	-215.11	0.30	-209.60	0.33	-220.36	0.22	-217.79	insufficient sampling
**1thg**	127-138	1.86	-1434.72	0.28	-1423.84	**1.12**	-1451.32	0.23	-1434.68	insufficient sampling / energy function deficiency
**2tgi**	48-59	3.14	-163.39	1.36	-87.48	0.41	-189.26	0.31	-182.44	insufficient sampling
**4i1b**	46-57	6.63	-140.98	1.04	-97.29	**6.64**	-140.95	0.81	-137.17	crystal packing

* bold numbers indicate cases where FKIC native input simulations also failed to correctly identify sub-Å models

Supplementary Table 6. *Multiple Segments* (12 residues) dataset detailed performance

		NGK					FKIC				
**PDB name**	Target segment residues	RMSD of lowest energy model (Å)	Lowest energy (REU)	Lowest RMSD (Å)	Energy of lowest RMSD model (REU)	Fraction sub-Å models	RMSD of lowest energy Model (Å)	Lowest energy (REU)	Lowest RMSD (Å)	Energy of lowest RMSD model (REU)	Fraction sub-Å models
**1ceo**	10-21, 53-64	3.74	-786.28	2.57	-763.87	0.00	2.47	-801.47	1.60	-774.43	0.00
**1deu**	47-58, 117-128	2.07	16572.20	1.27	16605.60	0.00	1.05	16537.10	0.53	16568.70	0.06
**1dqz**	146-157, 250-261	2.78	-657.72	0.89	-627.68	0.00	0.80	-656.04	0.80	-656.04	0.00
**1euv**	553-564, 527-538	1.35	-497.87	1.16	-480.99	0.00	2.59	-502.24	1.58	-495.34	0.00
**1ftr**	209-220, 276-287	9.25	-717.41	2.57	-698.25	0.00	7.33	-723.89	2.19	-708.61	0.00
**1h6u**	168-179, 190-201	1.01	-715.67	1.01	-715.67	0.00	0.40	-745.35	0.26	-733.33	0.64
**1i7k**	138-149, 66-77	1.80	-322.00	0.78	-312.85	0.00	0.47	-327.72	0.36	-321.18	0.30
**1j7d**	33-44, 67-78	2.30	-329.13	1.35	-323.97	0.00	1.98	-343.43	0.92	-333.10	0.01
**1jfu**	32-43, 134-145	5.49	-409.27	1.49	-391.57	0.00	1.29	-415.60	0.54	-400.58	0.05
**1ku1**	677-688, 729-740	4.37	567.65	1.65	576.01	0.00	1.11	560.84	0.67	563.89	0.02
**1m0z**	199-210, 244-255	3.35	-576.11	1.60	-126.88	0.00	2.17	-583.89	1.12	27.73	0.00
**1qs1**	264-275, 333-344	1.98	-957.12	1.77	-923.44	0.00	1.67	-970.92	0.90	-957.65	0.00
**1ryl**	54-65, 118-129	2.19	-363.80	1.73	-334.16	0.00	2.23	-362.49	1.75	-348.55	0.00
**1suu**	741-752, 780-791	6.21	-711.24	2.66	-698.90	0.00	3.11	-708.22	2.53	-692.72	0.00
**1t6g**	195-206, 220-231	1.10	-765.81	0.85	-747.86	0.01	1.87	-771.86	0.62	-756.32	0.17
**1u09**	238-249, 291-302	2.59	-945.68	1.25	-916.78	0.00	2.91	-952.12	0.60	-929.50	0.00
**1v5d**	282-293, 308-319	6.08	-938.87	4.75	-895.23	0.00	7.20	-945.93	5.15	-920.17	0.00
**1w0d**	65-76, 274-285	5.47	-661.63	1.25	-534.61	0.00	0.65	-676.38	0.31	-650.39	0.44
**1wko**	38-49, 109-120	1.52	-353.30	0.97	-321.36	0.00	1.19	-357.76	0.46	-321.97	0.08
**1xwt**	367-378, 38-49	5.47	-974.75	1.52	-954.86	0.00	2.62	-995.65	1.07	-950.23	0.00
**1xyz**	792-803, 813-824	7.55	-786.12	3.18	-737.98	0.00	1.99	-800.04	0.65	-767.73	0.00
**1yif**	142-153, 174-185	5.15	-773.41	2.34	-712.48	0.00	3.84	-781.24	1.91	-702.07	0.00
**1zvt**	657-668, 714-725	0.35	-545.67	0.32	-545.44	0.03	1.44	-542.72	1.41	-526.26	0.00
**2ahf**	182-193, 208-219	6.52	-707.08	3.03	-269.19	0.00	4.51	-775.93	0.80	-371.41	0.00
**2b49**	674-685, 652-663	0.55	-574.78	0.55	-574.78	0.04	2.88	-579.10	0.81	-572.12	0.00
**2c61**	410-421, 164-175	3.31	-981.07	0.74	-973.54	0.00	5.14	-987.68	1.29	-970.39	0.00
**2cyg**	248-259, 294-305	0.72	-728.09	0.72	-728.09	0.02	1.22	-753.92	0.58	-723.71	0.55
**2e01**	87-98, 256-267	2.02	-909.67	1.61	-894.78	0.00	0.44	-946.66	0.31	-922.67	0.75
**2g30**	790-801, 822-833	1.45	-500.35	1.18	-486.01	0.00	3.35	-486.53	1.61	-483.88	0.00
**2hjv**	299-310, 326-337	1.78	-390.05	1.08	-377.63	0.00	1.44	-393.43	1.35	-385.84	0.00

Supplementary Table 7. *Multiple Segments* (8 residues) dataset detailed performance

		NGK					FKIC				
**PDB name**	Target segment residues	RMSD of lowest energy model (Å)	Lowest energy (REU)	Lowest RMSD (Å)	Energy of lowest RMSD model (REU)	Fraction sub-Å models	RMSD of lowest energy model (Å)	Lowest energy (REU)	Lowest RMSD (Å)	Energy of lowest RMSD model (REU)	Fraction sub-Å models
**1a8d**	278-285,303-310	1.15	-998.03	0.35	-993.09	0.10	0.94	-983.98	0.73	-978.66	0.01
**1a8u**	225-232,251-258	0.94	-1073.51	0.94	-1073.51	0.00	0.65	-1081.86	0.58	-1036.74	0.42
**1ako**	150-157,173-180	0.45	-668.54	0.26	-666.92	0.66	0.43	-670.50	0.29	-662.28	0.68
**1bhe**	282-289,344-351	0.51	-877.73	0.28	-837.52	0.93	0.68	-875.49	0.38	-865.22	0.24
**1bn8**	250-257,338-345	1.98	-963.94	0.62	-959.38	0.05	2.45	-967.40	0.68	-949.27	0.00
**1brt**	28-35,205-212	0.76	-510.31	0.73	-500.12	0.21	0.81	-609.85	0.33	-488.96	0.94
**1c5e**	49-56,97-104	0.45	-702.21	0.29	-695.39	0.66	0.44	-703.36	0.26	-697.43	0.67
**1cil**	39-46,80-87	0.57	178.15	0.46	199.82	0.96	0.64	187.24	0.38	217.96	1.00
**1cs6**	126-133,158-165	1.10	-741.75	0.33	-719.69	0.57	0.72	-742.82	0.43	-741.61	0.73
**1dqz**	135-142,111-118	0.23	-1264.84	0.21	-1221.96	0.98	0.23	-1262.80	0.21	-1149.68	0.99
**1ede**	52-59,283-290	0.79	-689.00	0.69	-680.74	0.46	0.47	-694.65	0.32	-684.81	0.84
**1exm**	305-312,255-262	0.95	-998.75	0.30	-980.32	0.22	0.38	-994.71	0.31	-970.07	0.57
**1gai**	282-289,401-408	0.65	-306.61	0.26	-292.27	0.25	0.33	-294.19	0.28	-288.39	0.63
**1gof**	7-14,31-38	0.80	-1253.43	0.72	-1216.17	0.22	0.87	-1228.70	0.66	-1174.50	0.41
**1jev**	451-458,147-154	0.79	-1258.90	0.43	-1242.82	0.86	0.88	-1247.44	0.37	-1239.03	0.97
**1ms9**	433-440,468-475	2.27	-2629.19	0.32	-2392.50	0.14	2.74	-2411.81	0.29	-2332.46	0.02
**1nif**	64-71,221-228	1.49	-722.30	0.93	-643.85	0.00	1.50	-726.32	0.51	-651.66	0.14
**1oth**	219-226,280-287	0.30	-671.55	0.22	-640.91	1.00	0.27	-654.61	0.22	-649.22	1.00
**1qlw**	130-137,67-74	0.40	-1600.69	0.25	-1560.94	1.00	0.37	-1596.93	0.26	-1592.87	1.00
**1srp**	260-267,294-301	0.26	-760.01	0.19	-758.04	0.55	0.25	-760.73	0.19	-758.14	0.45
**1tad**	109-116,159-166	0.65	-1951.28	0.36	-1945.22	0.98	0.64	-1964.78	0.33	-1947.35	0.76
**1thg**	351-358,307-314	2.63	-979.60	2.07	-928.81	0.00	3.48	-977.31	1.80	-935.77	0.00
**1tib**	171-178,211-218	1.08	-466.52	0.67	-440.39	0.37	0.54	-471.50	0.29	-460.77	0.53
**1udc**	32-39,78-85	0.62	-668.79	0.54	-660.04	0.06	0.59	-667.57	0.46	-660.73	0.19
**3bto**	256-263,280-287	1.15	-3377.16	0.29	-3110.83	0.19	1.07	-3405.40	0.34	-3274.16	0.06
**3grs**	131-138,292-299	1.06	-998.99	0.29	-916.54	0.34	1.52	-1000.24	0.30	-929.86	0.67
**4pga**	112-119,216-223	0.60	-1315.25	0.24	-1309.43	0.97	0.57	-1380.06	0.26	-1313.87	1.00
**6cel**	132-139,367-374	1.36	-588.32	0.97	-568.66	0.00	1.70	-584.39	0.81	-573.68	0.00

Supplementary Table 8. Parameters of designs selected for experimental testing.

	Design Name	*Sequence Cluster*	*Struct Cluster*	*Largest Restraint Dist (Å) in lowest scoring model*	*Loop RMSD (Å)*	*Score Gap (REU)*	*% Sub-Å Restraints*	*% Sub-Å Loops*
**Full-length**	V1D1r	1	1	0.83	0.20	3.16	7.60	13.00
	V1D2r	1	1	1.05	0.38	2.05	0.20	12.20
	V1D3r	2	1	0.55	0.20	6.50	7.20	4.60
	V1D4	2	1	0.68	0.24	6.56	6.60	5.20
	V1D5r	2	1	0.92	0.27	11.88	2.20	2.00
	V1D6	3	2	2.37	0.33	0.00	0.00	17.20
	V1D7	3	3	2.63	0.25	0.00	0.00	3.20
**Del1**	V1D8r	1	2	0.57	0.09	4.72	11.20	31.80
	V1D9r	1	2	0.80	0.08	5.09	11.40	20.00
	V1D10r	1	2	0.87	0.18	4.51	13.20	19.40
	V1D11	2	1	2.25	0.21	0.00	0.60	10.60
	V1D12r	2	1	2.35	0.32	0.00	3.20	20.40
	V1D13	2	1	2.36	1.22	0.00	1.40	6.80
	V1D14	3	3	2.31	0.23	0.00	0.00	4.60
**Full-length**	V2D1	3	2	0.67	0.32	8.16	5.80	7.25
	V2D2	3	2	0.83	0.73	8.97	4.23	7.04
	V2D3	3	2	0.91	0.38	5.54	8.45	14.08
	V2D4	3	2	0.71	0.23	5.35	5.88	5.88
	V2D5	3	2	0.72	0.24	10.31	1.41	1.41
	V2D6	1	4	0.95	0.16	0.90	19.12	63.24
	V2D7	5	1	1.03	0.61	5.52	5.71	32.86
	V2D8	5	1	1.05	0.81	2.92	5.88	35.29
	V2D9	2	3	1.15	0.13	0.22	4.42	71.46
	V2D10	1	1	0.86	0.71	0.09	8.57	61.10
	V2D11	1	1	0.87	0.66	12.02	26.70	54.92

Boxes on the left side indicate whether designs were based off of a full-length input structure or contained a 1-residue deletion (Del1) in the catalytic loop. Sequence cluster: Designs were clustered hierarchically (see **Supplementary Methods**) according to sequence distance, determined using the BLOSUM80 substitution matrix. Struct Cluster: Designs were clustered (see **Supplementary Methods**) according to the C/Cα/N/O RMSD for the positions where the backbone was remodeled. Largest Restraint Dist (Å) in lowest scoring model: The furthest distance between any of the atoms in the E38 carboxylate (Cδ, Oε1, or Oε2) and their target positions, for the lowest scoring loop modeling decoy. Loop RMSD (Å): The average RMSD in Å between loop modeling decoys and the input design structure. Score Gap: The difference in fa_attr score between the lowest scoring decoy that puts all of the atoms of the E38 carboxylate less that 1Å from their target positions, and the lowest scoring decoy that puts at least one atom of the E38 carboxylate more than 2Å from its target position. A score gap of 0 indicates that the lowest-scoring decoy was more than 2Å from its target position. % Sub-Å Restraints: The fraction of the loop modeling decoys that are predicted to position the all atoms of the Glu carboxylate less than 1Å from their target positions. % Sub-Å Loops: The fraction of loop modeling decoys that are predicted to position all backbone atoms (C/Cα/N/O) within 1Å RMSD of the input design structure.

Supplementary Table 9a. List of mutations for PIP version 1 designs. Designs appended with "r" indicate that mutations were reverted to the wild-type residue based on visual inspection; 8/14 designs from version 1 contained reversion mutations.

Design Name	***Mutations***
**V1D1r**	T35K, E37T, D38E, P39D, V40A, S42L, E43G, P44G, R45Y, S46Q, A50W, V74N, L115T
**V1D2r**	D33N, T35Y, D38E, P39S, V40A, S42Q, E43P, P44K, R45Y, S46W, A49D, E53K, S58Q, V74A, M112A, L115I, E118D
**V1D3r**	T35K, E37I, D38E, P39T, V40Q, G41Y, S42P, S46K, V74A, M112A
**V1D4**	F30Y, D32P, D33N, T35K, E37I, D38E, P39T, V40Q, G41Y, S42P, S46K, T48R, A49D, A50N, N57E, S58A, K60R, V74A, A75N, N76G, V109I, V110N, S111Y, M112A, R113Q, L115V, E118P
**V1D5r**	D33N, T35R, D38E, P39T, V40K, G41Y, S42P, P44D, S46K, S58Q, K60A, V74A, M112A, R113Q
**V1D6**	F30Y, A31D, D33T, T35R, V36R, E37N, D38E, P39I, V40G, S42P, E43P, R45L, S46P, T48R, A49D, A50N, E53K, N57E, S58D, K60A, V74T, A75N, N76G, V109I, V110S, S111Y, M112A, R113Q, L115V, E118D
**V1D7**	F30Y, A31S, D32P, T35I, V36R, E37R, D38E, P39R, V40Y, G41A, S42K, E43A, P44N, R45P, S46R, T48R, A49D, E53Q, N57E, S58D, K60A, V74T, A75N, N76G, V109I, V110A, S111E, M112A, R113Q, L115I, E118D
**V1D8r**	F30Y, D32S, E37T, D38E, P39S, V40F, G41-, S42R, E43P, R45F, S46T, A49E, V74A, A75N, N76G, V109I, S111Y, M112A, R113Q
**V1D9r**	E37R, D38E, P39S, V40F, G41-, S42R, E43P, R45F, S46T, S58N, V74S, A75N, S111E, M112A, R113Q
**V1D10r**	T35S, E37T, D38E, P39S, V40F, G41-, S42R, E43P, R45F, S46T, S58N, V74S, A75N, M112A
**V1D11**	F30Y, D32P, D33N, T35R, V36Y, E37D, D38E, P39I, V40G, G41-, S42F, E43P, P44D, R45T, S46G, A49D, A50N, E53A, N57K, S58Q, V74T, A75N, N76G, V109I, V110A, S111E, M112A, L115I, E118D
**V1D12r**	T35Q, V36Y, E37N, D38E, P39I, V40G, G41-, S42F, E43R, P44G, R45D, S58Q, A75N, N76G, S111Y, M112A
**V1D13**	F30Y, A31D, D32S, D33T, T35Q, V36Y, E37D, D38E, P39I, V40G, G41-, S42F, E43D, P44G, R45G, A49E, A50N, E53R, N57K, S58Q, V74T, A75N, N76G, V109I, V110A, S111E, M112A, L115I, E118D
**V1D14**	F30Y, A31D, D32S, D33T, T35I, V36R, E37Y, D38E, P39Q, V40Y, G41-, S42Y, P44G, R45G, S46K, A49D, A50N, E53K, N57M, S58D, V74T, A75N, N76G, V109I, V110A, S111E, M112A, L115I, E118D

Supplementary Table 9b. List of mutations for PIP V2 designs. Designs appended with "r" indicate that mutations were reverted to the wild-type residue based on visual inspection; 11/22 designs from version 2 contained reversion mutations.

Design Name	*Mutations*
**V2D1**	V27A, A28G, L29F, F30L, D33G, A34I, T35K, V36I, E37D, D38E, P39D, V40Q, G41N, S42R, E43K, P44Q, R45V, S46T, G47D, T48A, A50Q, I51K, A73S, A75S
**V2D1r**	D33G, A34I, V36I, E37D, D38E, P39D, G41N, S42R, E43K, P44Q, R45V, G47D, A50Q, A73S, A75S
**V2D2**	V27A, A28G, L29F, F30L, D33G, A34V, T35K, E37D, D38E, P39D, V40Q, G41K, S42K, E43T, P44T, R45V, S46T, G47D, T48A, A50Q, I51K, A73S, A75D
**V2D2r**	D33G, A34V, E37D, D38E, P39D, V40Q, G41K, S42K, E43T, P44T, R45V, G47D, A50Q, A75D
**V2D3**	V27A, A28G, L29F, F30L, D33G, A34V, T35Q, V36I, E37D, D38E, P39D, V40Q, G41N, S42K, E43T, P44T, R45V, S46T, G47D, T48A, A50Q, I51K, A73S, A75D
**V2D3r**	D33G, A34V, V36I, E37D, D38E, P39D, V40Q, G41N, S42K, E43T, P44T, R45V, G47D, A50Q, A75D
**V2D4**	V27A, A28G, L29F, F30L, D33G, A34V, T35Q, V36I, E37D, D38E, P39D, V40Q, G41Q, S42T, E43S, P44T, R45V, S46T, G47D, T48A, A50Q, I51K, A73S, A75K
**V2D4r**	D33G, A34V, V36I, E37D, D38E, P39D, V40Q, G41Q, S42T, E43S, P44T, R45V, G47D, A50Q, A75K
**V2D5**	V27A, A28G, L29F, F30L, D33G, A34V, V36I, E37L, D38E, P39D, V40Q, G41Q, S42K, E43S, P44T, R45V, S46T, G47D, T48A, A50Q, I51K, A73S, A75D
**V2D5r**	D33G, A34V, V36I, E37D, D38E, P39D, V40Q, G41Q, S42K, E43S, P44T, R45V, G47D, A50Q, A75D
**V2D6**	F30L, D32S, A34V, V36L, E37W, D38E, P39T, V40S, G41Q, S42D, E43R, P44T, R45Y, S46T, T48N, A49S, A73S, V74N, A75R
**V2D6r**	V36L, E37W, D38E, P39T, V40S, G41Q, S42D, E43R, P44T, R45Y, V74N, A75R
**V2D7**	A28G, L29Q, A31G, D32P, D33Q, A34V, V36I, D38E, P39S, V40K, G41F, S42P, E43P, P44A, R45D, S46P, G47D, T48L, A49S, A73V, V74Y, A75N, N76Y, E77T, A78T
**V2D7r**	A31G, D32P, A34V, V36I, D38E, P39S, V40K, G41F, S42P, E43P, P44A, R45D, S46P, G47D, V74Y, A75N
**V2D8**	A28G, L29Q, A31G, D32P, D33Q, A34V, T35V, V36I, D38E, P39S, V40K, G41Q, S42P, E43P, P44T, R45D, S46P, G47D, T48L, A49S, A73V, V74Y, A75N, E77T, A78T
**V2D8r**	A31G, D32P, V36I, D38E, P39S, V40K, G41Q, S42P, E43P, P44T, R45D, S46P, G47D, V74Y, A75N
**V2D9**	F30L, D32S, A34V, V36L, E37Y, D38E, P39T, V40S, G41Q, S42D, E43R, P44T, R45Y, S46T, T48N, A49S, A73S, V74N, A75R, E77T
**V2D9r**	E37Y, D38E, P39T, V40S, G41Q, S42D, E43R, P44T, R45Y, S46T, V74N, A75R
**V2D10**	A28G, L29Q, A31G, D32P, D33Q, A34V, V36L, E37V, D38E, P39S, V40K, A(G)41S, S42P, E43P, P44A, R45D, S46P, A(G)47D, T48L, A49S, A73V, V74S, A75Q, E77T, A78T
**V2D11**	A28G, L29Q, A31G, D32P, D33Q, A34V, V36L, E37W, D38E, P39S, V40K, G41Y, S42P, E43P, P44A, R45D, S46P, G47D, T48L, A49S, A73V, V74S, A75G, A78T
**V2D11r(1)**	A31G, D32P, A34V, V36L, E37W, D38E, P39S, V40K, G41Y, S42P, E43P, P44A, R45D, S46P, G47D, T448L, A49S, V74S, A75G
**V2D11r(2)**	A31G, D32P, A34V, E37W, D38E, P39S, V40K, G41Y, S42P, E43P, P44A, R45D, S46P, G47D, T448L, A49S, V74S, A75G

Supplementary Table 10: Experimental characterization of designs from PIP version 1.

Design	Mutations	Expression	Purified	Solubility	Activity
**V1D1r**	14	Insoluble	Yes	Soluble	-
**V1D2r**	18	Insoluble	Yes	Insoluble	N/A
**V1D3r**	11	Insoluble	Yes	Soluble	-
**V1D4**	28	Insoluble	Yes	Insoluble	N/A
**V1D5r**	15	Insoluble	Yes	Insoluble	N/A
**V1D6**	31	None	No	N/A	N/A
**V1D7**	32	Insoluble	No	N/A	N/A
**V1D8r**	19	Insoluble	Yes	Soluble	+
**V1D9r**	15	Insoluble	Yes	Soluble	+
**V1D10r**	14	Insoluble	Yes	Soluble	+
**V1D11**	29	Insoluble	Yes	Insoluble	N/A
**V1D12r**	16	None	No	N/A	N/A
**V1D13**	29	Insoluble	Yes	Insoluble	N/A
**V1D14**	29	None	No	N/A	N/A

Mutations: Number of mutations from wild-type KSI, excluding deletions. Expression: Whether the design expressed in inclusion bodies (“Insoluble”) or not at all (“None”). Purified: Whether or not the design was successfully purified. Solubility: Whether the purified design was soluble after re-folding from inclusion bodies. Activity: Whether the design had any observable enzymatic activity (“+”) or not (“-”); N/A: not applicable as protein could not be purified or was not soluble after purification.

Supplementary Table 11. Comparison of median RMSD of lowest energy models on perturbed structures. References for the different methods are indicated. Bold numbers denote best performance for given dataset (excluding FKIC with homologous fragments).

Method	8 residue side chain perturbed (Å)	12 residue side chain perturbed (Å)	template based models (Å)	12 residue backbone perturbed (Å)	12 residue backbone perturbed no unavoidable clashes (Å)	12 residue backbone perturbed unavoidable clashes (Å)
**HLP[20] ***	2.2	2.25	-	-	-	-
**HLP-SS[4] ***	0.85	1.15	-	-	-	-
**NGK[2] ***	**0.4**	0.75	3.9	-	-	-
**Galaxy-PS1[26] ***	1.45	3.05	3.5	-	-	-
**Galaxy-PS2[24] ***	1.05	1.55	**3.3**	**1.65**	1.4	**1.8**
**FKIC**	0.45	**0.64**	3.9	1.68	**1.28**	2.59
**FKIC with homologous fragments**	0.42	0.54	-	-	-	-

* Values reported by ref[24].

Supplementary Table 12. Summary of energy function comparisons

Dataset	Sampling method	Rosetta energy function	Median RMSD of lowest energy model (Å)	Median RMSD of lowest RMSD model (Å)	Median RMSD all models (Å)	Median sub-Å fraction	Median time (s)
Standard	NGK	talaris2013	0.74	0.37	2.71	11.40%	2281
Standard	NGK	ref2015	0.64	0.37	2.70	13.00%	3642
Standard	FKIC	talaris2013	0.70	0.36	1.19	44.89%	1646
Standard	FKIC	talaris2014	0.64	0.35	1.27	46.20%	1813
Standard	FKIC	ref2015	0.62	0.32	1.16	47.80%	3456
Mixed	NGK	talaris2013	1.94	0.45	4.66	1.90%	3788
Mixed	NGK	ref2015	1.07	0.45	4.65	1.15%	7341
Mixed	FKIC	talaris2013	0.61	0.34	1.74	34.60%	3654
Mixed	FKIC	ref2015	0.53	0.34	1.46	52.30%	7196

Supplementary Table 13.

Structure	*V1D8r (6UAD)*	*V2D9r (6UAE)*
Wavelength	1.116Å	1.116Å
Resolution Range	46.03-1.75 (1.80-1.75)	105.00-1.93 (1.96-1.93)
Unit Cell	a=b=53.15Å , c=178.03Å<br>**α**=**β**=90° **γ**=120°	a=73.01Å , b=210.00Å , c=39.64Å<br>**α**=**β**=**γ**=90°
Space Group	$P6_522$	$P2_12_12$
Unique Reflections	15833 (1048)	46239 (2182)
Multiplicity	9.7 (3.9)	19.1 (17.7)
Completeness	99.2% (92.0%)	98.1% (94.2%)
$\langle I/\sigma I \rangle$	34.2 (4.0)	9.3 (1.0)
$CC_{1/2}$[27]	1.000 (0.939)	0.995 (0.652)
R_{pim}[16]	0.013 (0.159)	0.059 (0.818)
R_{work}[28]	0.1742 (0.2083)	0.1752 (0.3041)
R_{free}[28]	0.2095 (0.2852)	0.2122 (0.3916)
Total Refined Atoms	1244	4662
Protein Residues	121	495
Solvent Molecules	163	372
Refined Ligand Atoms	66	204
Average B-factor	24.8Å^2	37.7Å^2
$RMSD_{bonds}$	0.014Å	0.006Å
$RMSD_{angles}$	1.23°	0.91°
Rama. Plot:		
Favored	99.2%	99.0%
Allowed	0.8%	1.0%
Outliers	0.0%	0.0%
Molprobity Clashscore[7]	0.93	4.64
PDB ID	6UAD	6UAE

Supplementary Table 14. Cα distances between positions of designed residues in the crystal structures of V2D9r and WT.

Residue #	WT amino acid	V2D9r amino acid	Cα RMSD
**34**	ALA	ALA	0.17
**35**	THR	THR	0.26
**36**	VAL	VAL	0.32
**37**	GLU	TYR	0.48
**38**	ASN	GLU	1.31
**39**	PRO	THR	2.47
**40**	VAL	SER	5.96
**41**	GLY	GLN	6.55
**42**	SER	ASP	0.79
**43**	GLU	ARG	2.47
**44**	PRO	THR	0.91
**45**	ARG	TYR	0.50
**46**	SER	THR	0.22
**74**	VAL	ASN	0.63
**75**	ALA	ARG	1.02

SUPPLEMENTARY FIGURES

Supplementary Figure 1. Detailed FKIC protocol.

The FKIC / LHKIC modeling protocol has a build stage (yellow), a centroid sampling stage (light red) and a full atom sampling stage (red). Both the centroid stage and the full atom stage perform simulated annealing which ramp the *rama* and *fa_rep* terms of the Rosetta energy function[25,29] in outer cycles and ramp the temperature in inner cycles.

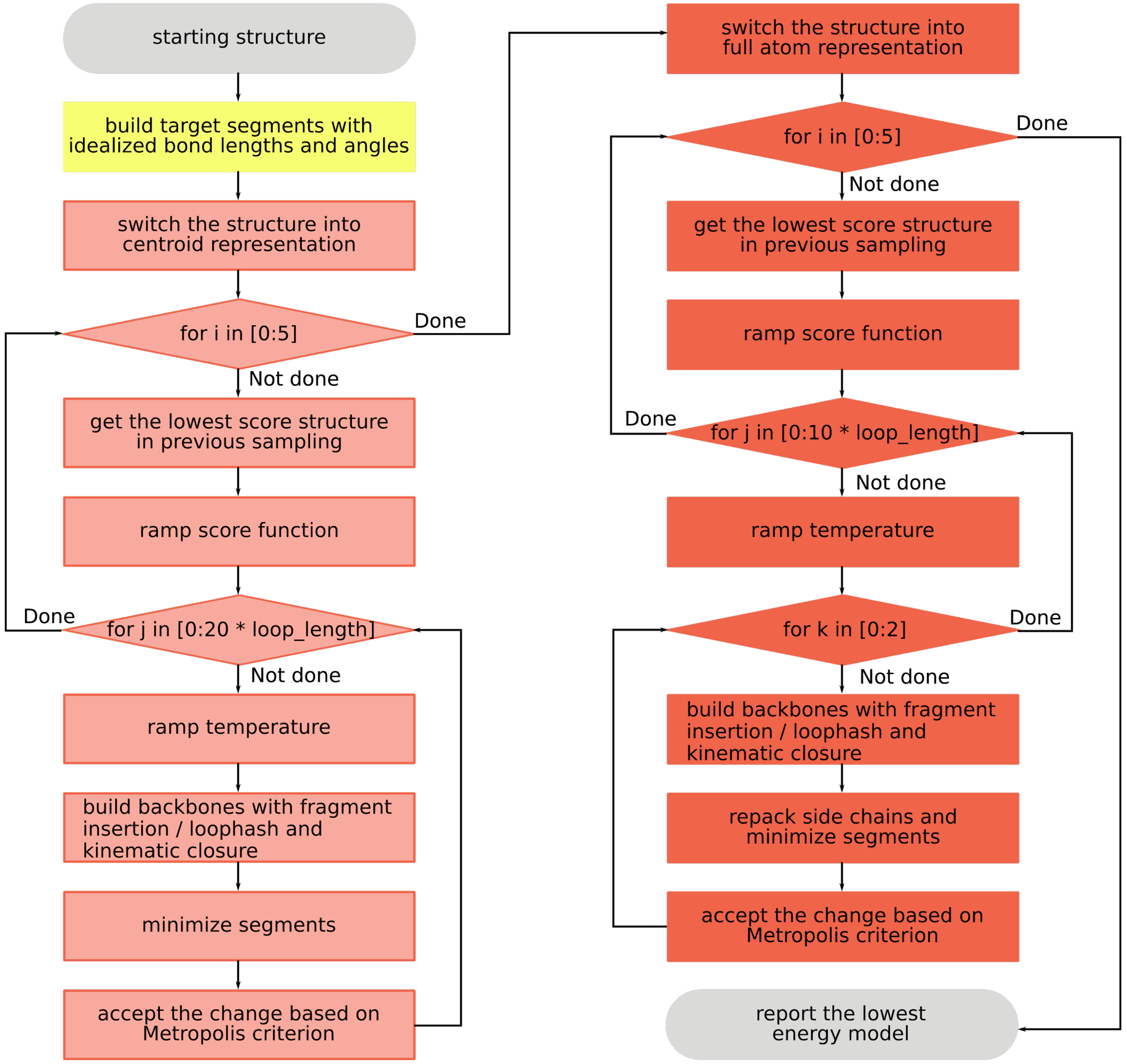

Supplementary Figure 2. Examples of failures of FKIC.

Results of standard FKIC are shown in red (right in each panel) and results of FKIC with native input information and native bond lengths and angles are shown in green (left in each panel). Each point represents a Rosetta generated model. REU, Rosetta energy units. (**a**) Sub-Å models are generated only with native inputs. (**b**) Standard FKIC generates a few sub-Å models but they are not identified by energy. Using native inputs generates a larger number of near-native solutions that can be correctly identified by energy. (**c**) The simulation with native inputs correctly identifies native-like models, but a model generated by standard FKIC has lower energy. (**d**) Neither standard nor native-input simulations correctly identify sub-Å models.

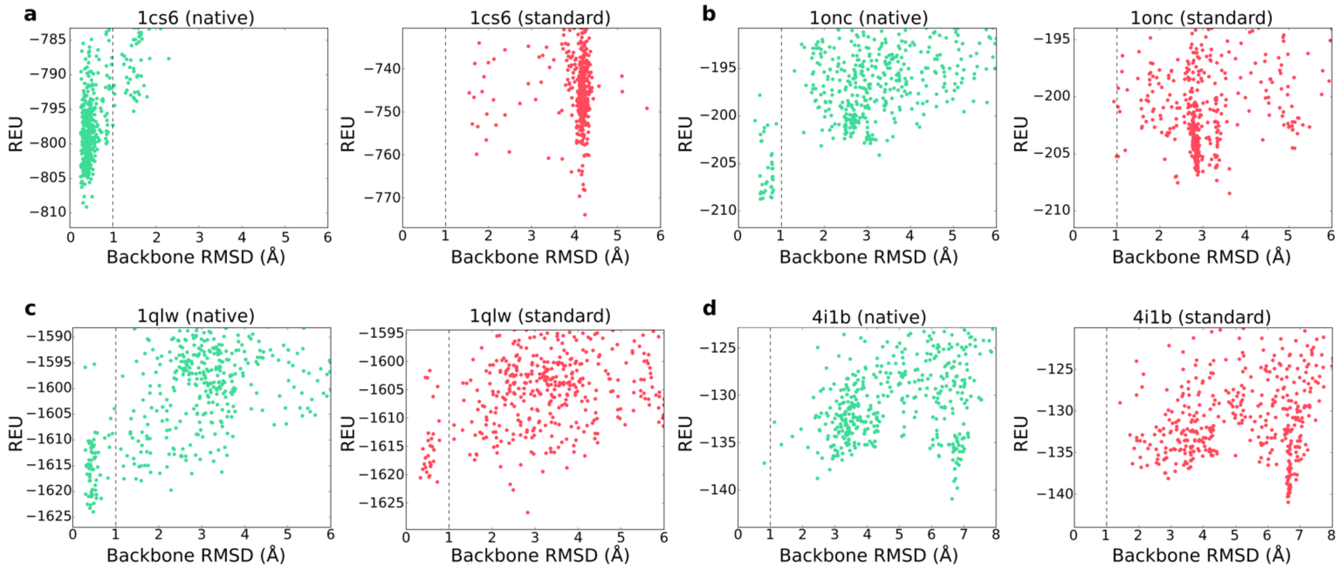

Supplementary Figure 3 Computational structure prediction of designs from PIP V1.

Rosetta total score (in REU) versus loop backbone RMSD (in Å) for designs selected for experimental testing from PIP version 1. Vertical dashed lines indicate 1Å loop RMSD. The experimentally characterized V1D8r design is highlighted in yellow. Structure prediction was performed on both the initial designs and the reversion mutants.

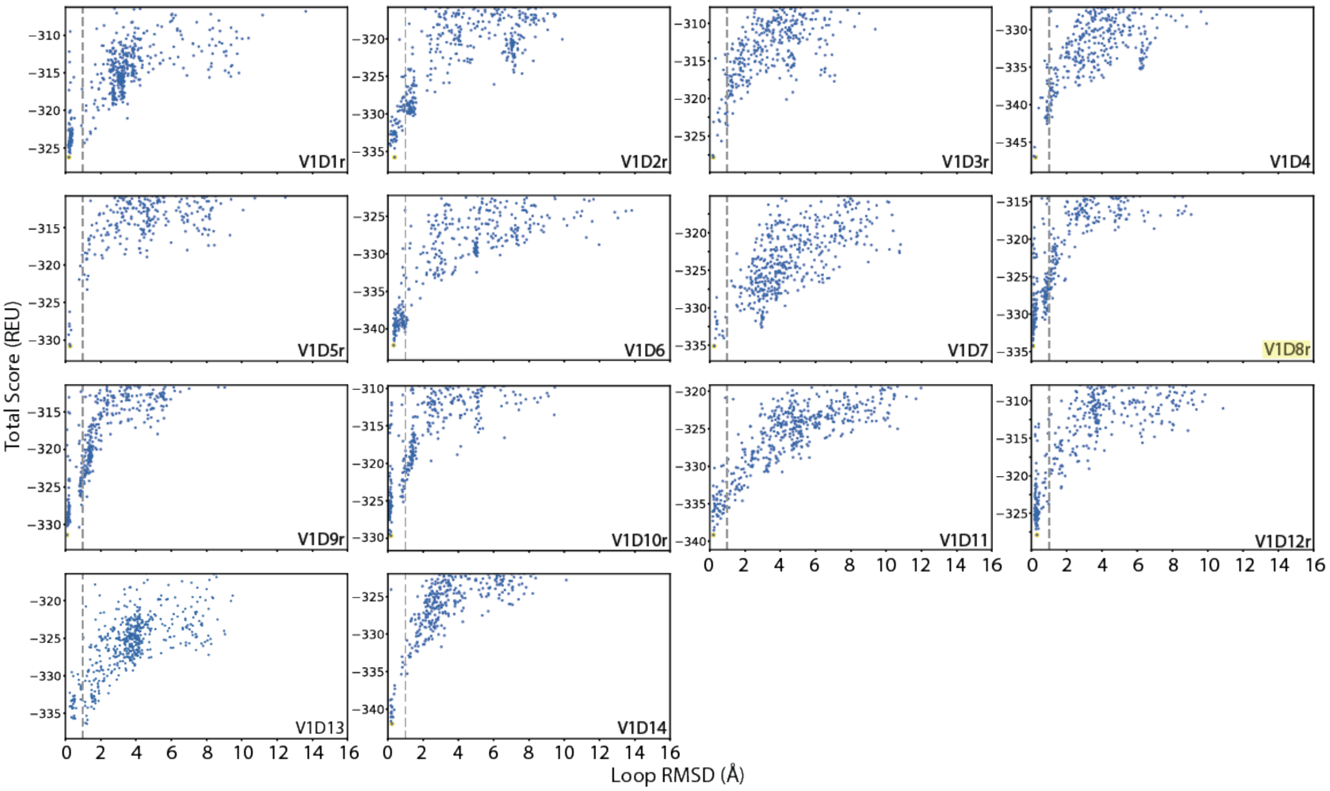

Supplementary Figure 4. Computational structure predictions of designs from PIP V2. Rosetta total score (in REU) versus loop backbone RMSD (in Å) for designs selected for experimental testing from PIP version 2. Plots are shown for the designs excluding the reversion mutants. Vertical dashed lines indicate 1Å loop RMSD. V2D9, the design corresponding to the experimentally characterized V2D9r reversion mutant, is highlighted in yellow.

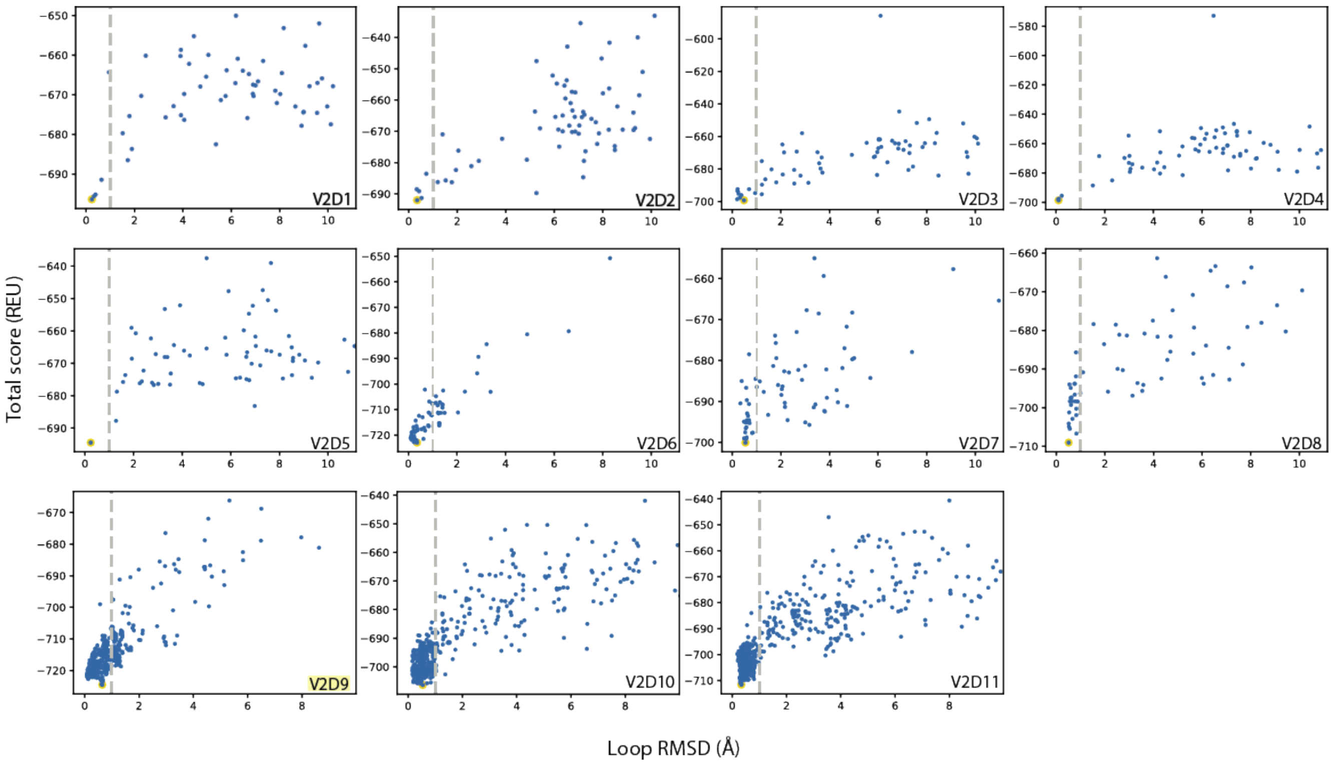

Supplementary Figure 5. Biophysical and biochemical characterization of designs.

Circular dichroism (CD) spectra for wild-type (**a**), V1D8r (**b**), or V2D9r (**c**). (**d**) Normalized temperature melting curves, measured via CD at 222 nm, for V1D8r (dark blue) and V2D9r (dark red) and their corresponding E38D reversion mutants (light blue and orange, respectively). The temperature melts are not reversible. Apparent melting temperatures are as follows: V1D8r: 44 °C, V1D8r E38D: 43 °C, V2D9r: 67 °C, V2D9r E38D: 67 °C. (**e**) Normalized absorbance (280 nm) from analytical size exclusion chromatography of V1D8r, V2D9r, wild-type KSI or a standards mixture with molecular weights as labeled.

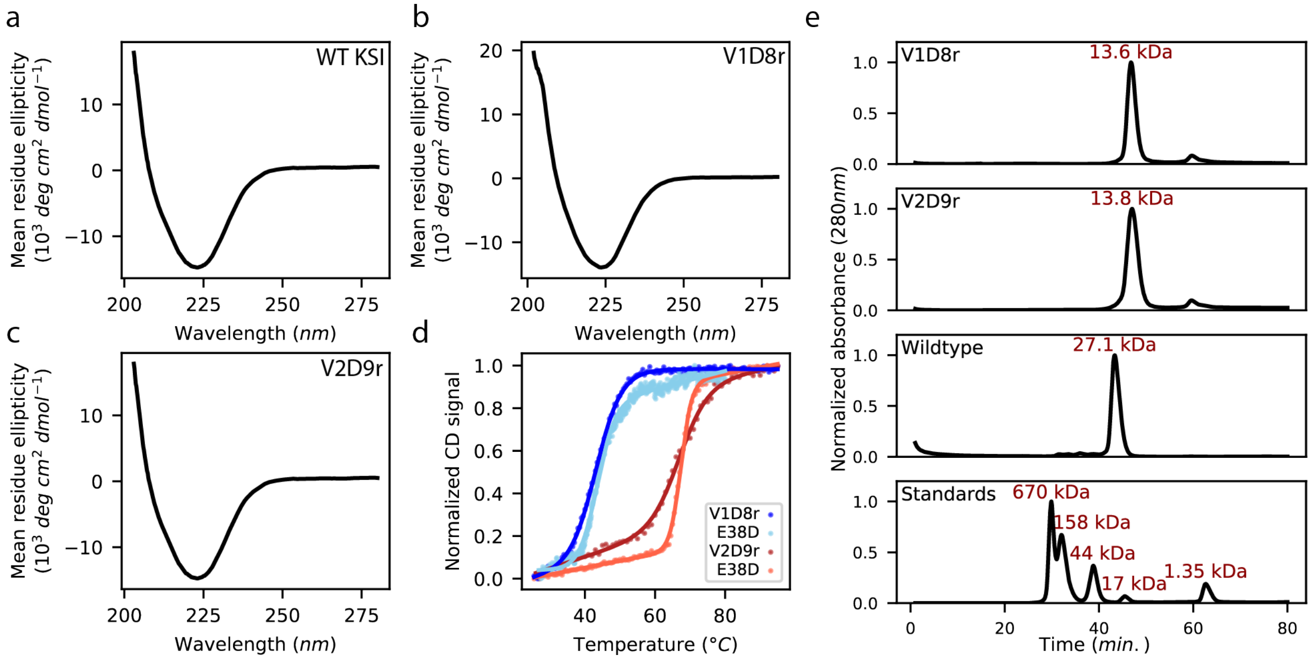

Supplementary Figure 6. Structural analysis of designs.

Electron density of reshaped backbone region for V1D8r **(a)** and V2D9r **(b)** at 1.0 sigma in mesh representation. **(c)** Electron density of possible alternate conformations of E38 observed in design 2. Density is contoured at 0.5 sigma for residues 37-39 in chain B. Residue E38 (teal) and equilenin (purple) are shown as sticks. Comparison of buried (<40 Å^2 solvent-accessible surface area) sidechain positioning between lowest-energy design model (orange) and crystal structure (blue) for **(d)** V1D8r or **(e)** V2D9r. Heavy-atom RMSDs for the displayed residues are shown in each panel. **(f)** Cα RMSD of the closest 9-residue fragment whose midpoint (5th residue) corresponds to the x-axis position. Top: Fragments picked using the WT sequence (blue) or the incorrect V2D9r sequence (red) aligned to the corresponding position in the WT crystal structure. Bottom: Fragments picked using the V2D9r sequence (blue) or the incorrect WT sequence (red) aligned to the corresponding position in the V2D9r crystal structure. The difference in fragment Cα RMSD between the correct and incorrect sequences are shown at the bottom of each graph. Blue bars (negative change in fragment Cα RMSD) indicate the correct structure is favored.

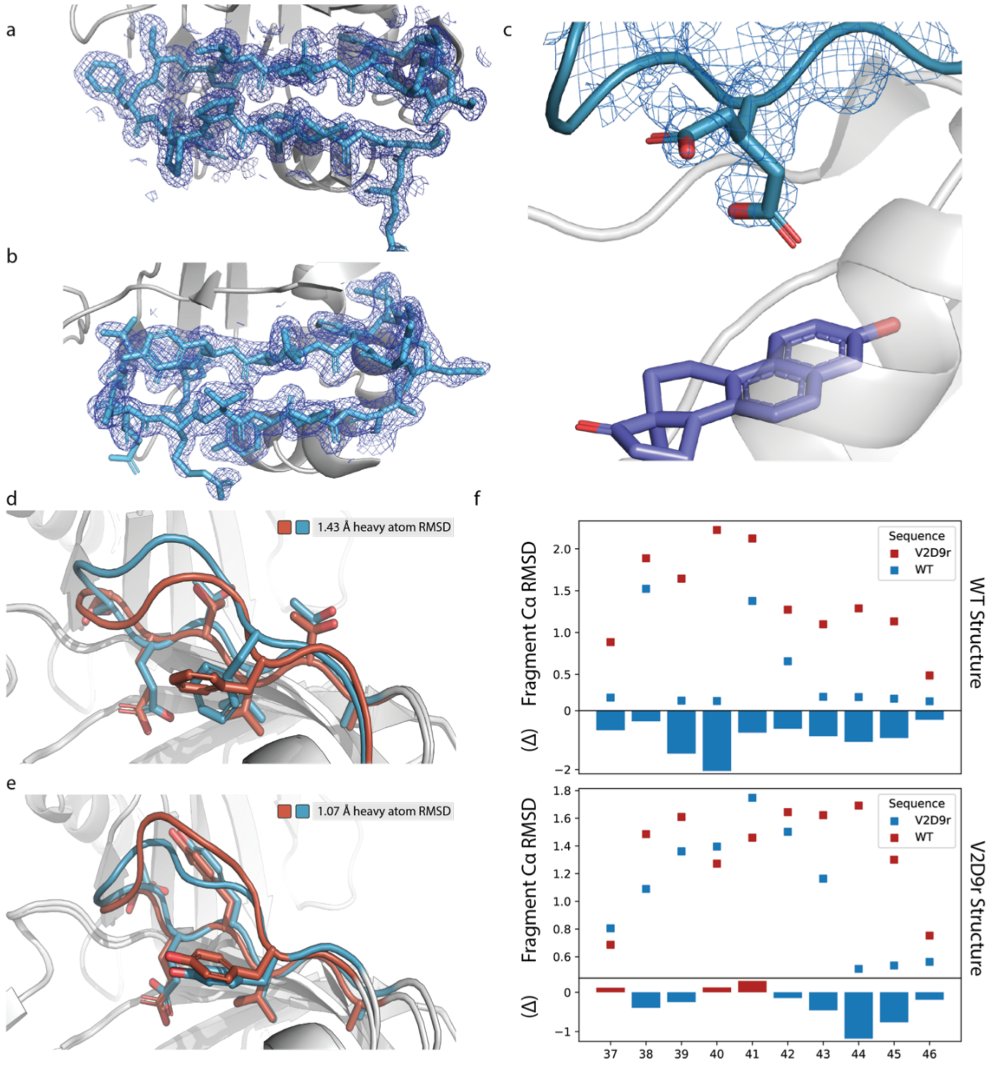

Supplementary Figure 7. Selection of designs for via Pareto fronts in PIP version 2.

Designs picked for computational structure prediction. Plots of design Lennard-Jones attractive (fa_attr) Rosetta score, in REU, vs. restraint satisfaction (longest distance of any restrained atom to its ideal position, in Å (**Supplementary Methods**)) for the first (**a**), second (**b**), and third (**c**) iterations of design for PIP version 2. Designs chosen via Pareto fronts for structure prediction are shown in red, other designs in blue.

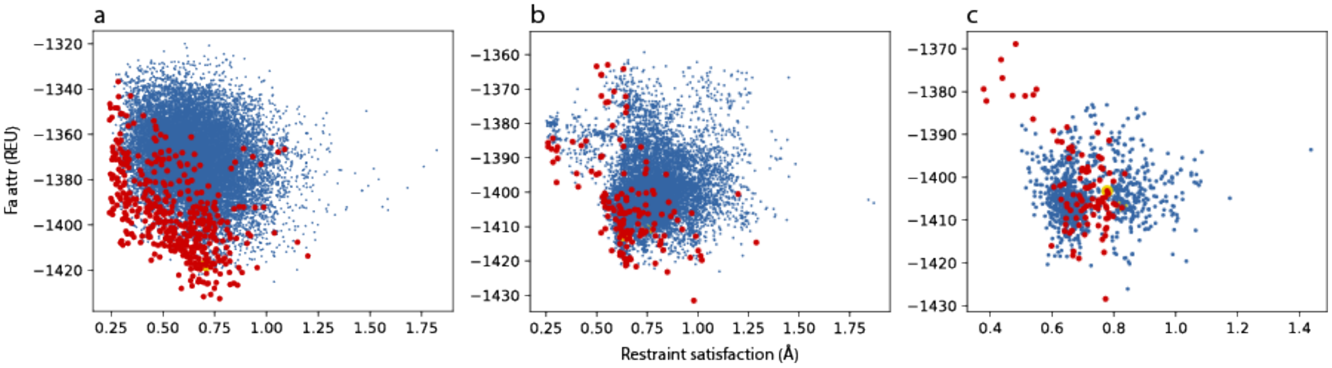

REFERENCES cited in the Supplementary Materials

1. Mandell, D.J., Coutsias, E.A. & Kortemme, T. Sub-angstrom accuracy in protein loop reconstruction by robotics-inspired conformational sampling. *Nat Methods* **6**, 551-2 (2009).
2. Stein, A. & Kortemme, T. Improvements to robotics-inspired conformational sampling in rosetta. *PLoS One* **8**, e63090 (2013).
3. O. Conchuir, S. et al. A Web Resource for Standardized Benchmark Datasets, Metrics, and Rosetta Protocols for Macromolecular Modeling and Design. *PLoS One* **10**, e0130433 (2015).
4. Sellers, B.D., Zhu, K., Zhao, S., Friesner, R.A. & Jacobson, M.P. Toward better refinement of comparative models: predicting loops in inexact environments. *Proteins* **72**, 959-71 (2008).
5. Wang, C., Bradley, P. & Baker, D. Protein-protein docking with backbone flexibility. *J Mol Biol* **373**, 503-19 (2007).
6. Touw, W.G. et al. A series of PDB-related databanks for everyday needs. *Nucleic Acids Res* **43**, D364-8 (2015).
7. Chen, V.B. et al. MolProbity: all-atom structure validation for macromolecular crystallography. *Acta Crystallogr D Biol Crystallogr* **66**, 12-21 (2010).
8. Gront, D., Kulp, D.W., Vernon, R.M., Strauss, C.E. & Baker, D. Generalized fragment picking in Rosetta: design, protocols and applications. *PLoS One* **6**, e23294 (2011).
9. Altschul, S.F. et al. Gapped BLAST and PSI-BLAST: a new generation of protein database search programs. *Nucleic Acids Res* **25**, 3389-402 (1997).
10. Ward, J.J., McGuffin, L.J., Buxton, B.F. & Jones, D.T. Secondary structure prediction with support vector machines. *Bioinformatics* **19**, 1650-5 (2003).
11. Yang, Y., Faraggi, E., Zhao, H. & Zhou, Y. Improving protein fold recognition and template-based modeling by employing probabilistic-based matching between predicted one-dimensional structural properties of query and corresponding native properties of templates. *Bioinformatics* **27**, 2076-82 (2011).
12. Cho, H.S. et al. Crystal structure of delta(5)-3-ketosteroid isomerase from Pseudomonas testosteroni in complex with equilenin settles the correct hydrogen bonding scheme for transition state stabilization. *J Biol Chem* **274**, 32863-8 (1999).
13. Schwans, J.P. et al. Use of anion-aromatic interactions to position the general base in the ketosteroid isomerase active site. *Proc Natl Acad Sci U S A* **110**, 11308-13 (2013).
14. Rocklin, G.J. et al. Global analysis of protein folding using massively parallel design, synthesis, and testing. *Science* **357**, 168-175 (2017).
15. Kim, S.W. & Choi, K.Y. Identification of active site residues by site-directed mutagenesis of delta 5-3-ketosteroid isomerase from Pseudomonas putida biotype B. *J Bacteriol* **177**, 2602-5 (1995).
16. Winter, G. xia2: an expert system for macromolecular crystallography data reduction. *Journal of Applied Crystallography* **43**, 186-190 (2010).
17. Kabsch, W. Xds. *Acta Crystallogr D Biol Crystallogr* **66**, 125-32 (2010).
18. Evans, P. Scaling and assessment of data quality. *Acta Crystallogr D Biol Crystallogr* **62**, 72-82 (2006).
19. Rapp, C.S. & Pollack, R.M. Crystal packing effects on protein loops. *Proteins* **60**, 103-9 (2005).
20. Jacobson, M.P. et al. A hierarchical approach to all-atom protein loop prediction. *Proteins* **55**, 351-67 (2004).
21. Kim, D.H. et al. Equilibrium and kinetic analysis of folding of ketosteroid isomerase from Comamonas testosteroni. *Biochemistry* **39**, 13084-92 (2000).
22. Yabukarski, F. et al. Assessment of enzyme active site positioning and tests of catalytic mechanisms through X-ray-derived conformational ensembles. *Proc Natl Acad Sci U S A* **117**, 33204-33215 (2020).

23. Hawkinson, D.C., Eames, T.C. & Pollack, R.M. Energetics of 3-oxo-delta 5-steroid isomerase: source of the catalytic power of the enzyme. *Biochemistry* **30**, 10849-58 (1991).
24. Park, H., Lee, G.R., Heo, L. & Seok, C. Protein loop modeling using a new hybrid energy function and its application to modeling in inaccurate structural environments. *PLoS One* **9**, e113811 (2014).
25. Park, H. et al. Simultaneous Optimization of Biomolecular Energy Functions on Features from Small Molecules and Macromolecules. *J Chem Theory Comput* **12**, 6201-6212 (2016).
26. Park, H. & Seok, C. Refinement of unreliable local regions in template-based protein models. *Proteins* **80**, 1974-86 (2012).
27. Karplus, P.A. & Diederichs, K. Linking crystallographic model and data quality. *Science* **336**, 1030-3 (2012).
28. Brunger, A.T. Free R value: cross-validation in crystallography. *Methods Enzymol* **277**, 366-96 (1997).
29. Alford, R.F. et al. The Rosetta all-atom energy function for macromolecular modeling and design. *J Chem Theory Comput* (2017).
